# Constrained Total Generalized p-Variation Minimization for Few-View X-Ray Computed Tomography Image Reconstruction

**DOI:** 10.1371/journal.pone.0149899

**Published:** 2016-02-22

**Authors:** Hanming Zhang, Linyuan Wang, Bin Yan, Lei Li, Ailong Cai, Guoen Hu

**Affiliations:** National Digital Switching System Engineering and Technological Research Center, Zhengzhou, 450002, China; Chongqing University, CHINA

## Abstract

Total generalized variation (TGV)-based computed tomography (CT) image reconstruction, which utilizes high-order image derivatives, is superior to total variation-based methods in terms of the preservation of edge information and the suppression of unfavorable staircase effects. However, conventional TGV regularization employs *l*_1_-based form, which is not the most direct method for maximizing sparsity prior. In this study, we propose a total generalized p-variation (TGpV) regularization model to improve the sparsity exploitation of TGV and offer efficient solutions to few-view CT image reconstruction problems. To solve the nonconvex optimization problem of the TGpV minimization model, we then present an efficient iterative algorithm based on the alternating minimization of augmented Lagrangian function. All of the resulting subproblems decoupled by variable splitting admit explicit solutions by applying alternating minimization method and generalized p-shrinkage mapping. In addition, approximate solutions that can be easily performed and quickly calculated through fast Fourier transform are derived using the proximal point method to reduce the cost of inner subproblems. The accuracy and efficiency of the simulated and real data are qualitatively and quantitatively evaluated to validate the efficiency and feasibility of the proposed method. Overall, the proposed method exhibits reasonable performance and outperforms the original TGV-based method when applied to few-view problems.

## Introduction

X-ray computed tomography (CT) serves revolutionary functions in biology, medicine, and other fields. Considering that excessive X-ray radiation exposure may cause genetic disease, recent studies have aimed to reduce radiation dose during X-ray CT inspection [1–2]. A promising strategy to reduce radiation dose is to under-sample the X-ray projections across the human body. However, image reconstruction from few-views can be treated as an ill-posed mathematical problem that is difficult to converge to the correct solution without extra prior information.

The development of compressive sensing (CS) theory has spurred considerable research attention on the additional sparse prior of images to reduce the sampling rate [3–4]. Total variation (TV) regularization employing the image gradient sparsity is a popular method that handles few-views problems in CT image reconstruction [5–15]. However, TV is based on the assumption that the image is piecewise constant. Thus, the TV algorithm suffers from over-smoothing and staircase effect, which in turn may produce undesirable blocky images [16, 17].

Several methods have been proposed to improve the performance of TV and eliminate the above drawback [18–26]. To our knowledge, two strategies have been widely investigated: 1) improvement of the original TV norm by introducing a penalty weight with sufficient local information, such as the edge-preserving TV (EPTV) model [18] and the adaptive-weighted TV (AwTV) model [19]; 2) involvement of high-order derivatives [22–27], such as total variation stokes(TVS) model [24], the high-degree TV (HDTV) model [25], and the total generalized variation (TGV) model [26, 27], et al. The strategy of incorporating TV norm with local information can lower staircase effects but often still retain some artifacts. The latter strategy usually shows a favorable performance with a suitable balance between first-order and high-order derivatives. In particular, TGV regularization can automatically balance first-order and high-order derivatives instead of using any fixed combination [28]. Hence, this process can yield visually pleasant results in images with piecewise polynomial intensities and sharp edges without staircase effects.

In the traditional concept, TGV is based on the *l*_1_-norm, which is a relaxation of the *l*_0_-norm for easy computation at the expense of performance on employing sparsity prior. In fact, the most direct measure of sparsity is to count the nonzero components of the target vector [29]; this strategy leads to an *l*_0_-norm solution but encounters nondeterministic polynomial-time hard (NP-hard) problems. Employing an *l*_p_-norm (0 < *p* < 1) relaxation for convenient properties in sparsity seeking has gained considerable interest in recent years [30–33]. Sidky et al. replaced the *l*_1_-norm with the *l*_p_-norm in the minimization function and proposed a total p-variation (TpV) minimization algorithm [34]. Although the *l*_p_-norm causes nonconvex optimization problems, it may allow efficient image reconstruction with a few projection datasets for radiation dose reduction [35].

In this article, we explore an *l*_p_-norm (0 < *p* < 1) relaxation, which is close to the *l*_0_-norm and can accurately measure sparsity, to improve the sparsity seeking features of TGV. The proposed regularization model is called total generalized p-variation (TGpV). The proposed model is efficiently solved through variable splitting and alternating minimization method in conjunction with nonconvex p-shrinkage mapping [31]. The novelty of this work is threefold. First, the TGpV model is far less restrictive than the TGV and TpV models for X-ray CT image reconstruction. It not only shows excellent performance in detail preservation by incorporating high-order image derivatives but also achieves an accurate measurement of sparsity potential from image regularity prior. Second, an effective iterative algorithm is proposed to optimize the objective function of the TGpV minimization with a fast and stable convergence result. Third, fast and efficient closed-form solutions are investigated and derived for computationally complex subminimization problems by using the proximal point technique and fast Fourier transforms. The advantage of our approach is demonstrated in both numerical simulation and real CT data, relative to the previous TpV-based and TGV-based reconstructions.

The remainder of this paper is organized as follows. Section 2 briefly introduces the TGpV model and then describes the constrained TGpV minimization model and the present TGpV-ADM algorithm for image reconstruction. Section 3 presents the experimental results. Finally, Sections 4 and 5 respectively contain the discussions and conclusions.

## Methods

### Total Generalized p-Variation

Let *α* = (*α*_0_, *α*_1_) denote the positive weights, the discretized second-order TGV [28] can be written as follows:

$$\text{TG}{\text{V}}_{\alpha }^{2}\left(u\right)=\text{arg}\underset{\omega }{\text{min}}\alpha_{0}\|\nabla u-\omega {\|}_{1}+\alpha_{1}\|\varepsilon (\omega ){\|}_{1},$$ (1)

where *ω* is a variable used to balance the first and higher order derivatives, and the operators ▽ and *ε* are given by

$$\nabla u=\left[\begin{array}{l} \nabla_{x}u\\ \nabla_{y}u \end{array}\right],\;\varepsilon (\text{v})=\left[\begin{array}{l} \nabla_{x}v_{x}\;\frac{1}{2}(\nabla_{y}v_{x}+\nabla_{x}v_{y})\\ \frac{1}{2}(\nabla_{y}v_{x}+\nabla_{x}v_{y})\;\nabla_{y}v_{y}\; \end{array}\right].$$ (2)

The original version of TGV uses an *l*_1_-based form. The sparsity of a target vector is generally measured directly by counting the nonzero components in it; using an *l*_0_-norm solution is a better way to take advantage of the sparsity prior. However, this strategy involves an NP-hard problem and lacks efficient solvers for practical image reconstruction.

Another strategy is to using the *p*th power of the *l*_p_-norm (0 < *p* < 1) which is a relaxation closer to the *l*_0_-norm and can measure sparsity better than the *l*_1_-norm. Hence, dealing with *l*_p_ minimization, we propose the following modified form of the second-order TGpV:

$$\text{TGp}{\text{V}}_{\alpha }^{2}\left(u\right)=\text{arg}\underset{\omega }{\text{min}}\alpha_{0}\|\nabla u-\omega {\|}_{p}+\alpha_{1}\|\varepsilon (\omega ){\|}_{p},\;0<p<1.$$ (3)

The *l*_p_-norm based form of TGV can express a lower level of sparsity than the conventional form. Thus, maximizing this sparsity can further relax the requirements of data sampling. On the other hand, a multiple description of sparsity leads to wide selections and may provide a comprehensive validity for different objects.

### Constrained TGpV minimization

To promote the sparsity feature of TGpV, we introduce it into the CT imaging model based on a regularization framework. The CT imaging model can be approximated by the following discrete linear system:

b=Au. (4)

The vector *b* represents the projection data, the vector *u* represents the object to be reconstructed, and the system matrix *A* is a pixel-driven projection operator.

To solve the linear system of Eq (4), a constrained TGpV minimization model for describing the intensity variations of an image is used as follows:

$$\;u^{*}=\text{arg}\underset{u}{\text{min}}\text{TGp}{\text{V}}_{\alpha }^{2}\left(u\right),\;\text{subject}\;\text{to}\;\|Au-b{\|}_{2}\leq e,$$ (5)

where *e* is a data-error tolerance parameter.

The optimization problem in Eq (5) is referred to as TGpV minimization. In this study, we investigate CT image reconstruction by minimizing the energy function with the TGpV regularization term by solving the constrained nonconvex optimization problem as follows:

$$\text{arg}\underset{u,\omega }{\text{min}}\alpha_{0}\|\nabla u-\omega {\|}_{p}+\alpha_{1}\|\varepsilon (\omega ){\|}_{p},\;\text{s}.\text{t}.\;\|Au-b{\|}_{2}\leq e\;.$$ (6)

### TGpV minimization reconstruction algorithm

We apply variable splitting and alternating direction method (ADM) [36] to obtain efficient and easy-to-code algorithms for solving the minimization problems involved in our method. A generalized p-shrinkage operator [31] showing a qualitative resemblance to the *l*_p_ proximal mapping is considered to provide a closed solution to the *l*_p_—*l*_2_ problem in the reconstruction procedure. The reconstruction algorithm that utilizes TGpV minimization is summarized below.

Introducing the vectors *d*, *s* and *σ*, we consider the following constrained minimization problem, which is equivalent to Eq (6):

$$\begin{array}{l} \;\underset{u,\omega ,y,z}{\text{min}}\alpha_{0}\|d{\|}_{p}+\alpha_{1}\|s{\|}_{p},\\ s.t.\;\nabla u-\omega =d,\;\varepsilon (\omega )=s\;\text{and}\;Au-b=\sigma ,\;\|\sigma {\|}_{2}\leq e. \end{array}$$ (7)

To reformulate the original constrained problem to a sequence of unconstrained subproblems, the augmented Lagrangian method [37] is used here. The augmented Lagrangian energy associated to Eq (7) is defined as

$$\begin{array}{l} L_{\;A}(u,\omega ,d,s,\tilde{d},\tilde{s},\tilde{r})=\alpha_{0}\|d{\|}_{p}+{\tilde{d}}^{T}(d-\nabla u+\omega )+\frac{\lambda_{0}}{2}\|d-\nabla u+\omega {\|}_{2}^{2}\\ \;+\alpha_{1}\|s{\|}_{p}+{\tilde{s}}^{T}(s-\varepsilon (\omega ))+\frac{\lambda_{1}}{2}\|s-\varepsilon (\omega ){\|}_{2}^{2}\\ \;-{\tilde{r}}^{T}(Au-b-\sigma )+\frac{\mu }{2}\|Au-b-\sigma {\|}_{2}^{2}, \end{array}$$ (8)

where $\tilde{d},\tilde{s},\tilde{r}$ are Lagrange multipliers, and *λ*_0_, *λ*_1_, and *μ* are positive constants used to balance the terms.

As a powerful technique to optimize problems through variable splitting, the alternating direction method is used to solve the problem efficiently. The augmented Lagrangian function *L*_*A*_ can be split into four subproblems with respect to *d*, *s*, *u*, and *ω*. The solution to minimizing *L*_*A*_ is equivalent to solving the subminimization problems as follows:

i. The subminimization problem with respect to *d* can be written as follows:

$$\underset{d}{\text{min}}\alpha_{0}\|d{\|}_{p}+\frac{\lambda_{0}}{2}\|d+\frac{{\tilde{d}}^{k}}{\lambda_{0}}-\nabla u^{k}+\omega^{k}{\|}_{2}^{2}.$$ (9)

This minimization problem corresponds to an *ℓ*_*p*_−*ℓ*_2_ norm. To derive an efficient solution to this problem, an explicit proximal mapping for general *p* is considered [31]. The p-shrinkage operator *shrink*_*p*_ (·,1/*β*) is defined as

$$shrink_{p}(\xi ,1/\beta )≜\text{max}\left\{\left|\xi \right|-\beta^{p-2}|\xi {|}^{p-1},0\right\}\;\cdot \;\frac{\xi }{\left|\xi \right|}.$$ (10)

Thus, this minimization problem can be directly solved by

$$d^{k+1}=shrink_{p}(\nabla u^{k}-\omega^{k}-\frac{{\tilde{d}}^{k}}{\lambda_{0}},\alpha_{0}/\lambda_{0}).$$ (11)

ii. The minimization w.r.t *s* can be written as

$$\underset{s}{\text{min}}\alpha_{1}\|s{\|}_{p}+\frac{\lambda_{1}}{2}\|s+\frac{{\tilde{s}}^{k}}{\lambda_{1}}-\varepsilon (\omega^{k}){\|}_{2}^{2}.$$ (12)

This problem is solved with the same adaptation of the p-shrinkage operator as follows:

$$s^{k+1}=shrink_{p}(\varepsilon (\omega^{k})-\frac{{\tilde{s}}^{k}}{\lambda_{1}},\alpha_{1}/\lambda_{1}).$$ (13)

iii. The subminimization problem w.r.t *u* corresponds to the following quadratic positive definite problem:

$$\underset{u}{\text{min}}\frac{\mu }{2}\|Au-b-\sigma^{k}{\|}_{2}^{2}-{\tilde{r}}^{T}(Au-b-\sigma^{k})+\frac{\lambda_{0}}{2}\|d^{k+1}+\frac{{\tilde{d}}^{k}}{\lambda_{0}}-\nabla u+\omega^{k}{\|}_{2}^{2}.$$ (14)

The first-order necessary conditions for optimization are

$$(\mu A^{T}A+\lambda_{0}\nabla^{T}\nabla )u=\mu A^{T}(b+\sigma^{k})+A^{T}\tilde{r}+\lambda_{0}\nabla^{T}(d^{k+1}+\frac{{\tilde{d}}^{k}}{\lambda_{0}}+\omega^{k}).$$ (15)

The exact minimizer of Eq (14) is formulated as

$$u^{k+1}={\left(\mu A^{T}A+\lambda_{0}\nabla^{T}\nabla \right)}^{+}\left(\mu A^{T}(b+\sigma^{k})+A^{T}\tilde{r}+\lambda_{0}\nabla^{T}(d^{k+1}+\frac{{\tilde{d}}^{k}}{\lambda_{0}}+\omega^{k})\right),$$ (16)

where M+ stands for the Moore-Penrose pseudoinverse of matrix M.

Then, the noise term *σ* can be updated by

$$\sigma^{k+1}=\text{min}\left\{1,\;e/\|Au^{k+1}-b{\|}_{2}^{}\right\}\cdot \left(Au^{k+1}-b\right).$$ (17)

iv. The subminimization problem w.r.t *ω* can be written as follows:

$$\underset{\omega }{\text{min}}\frac{\lambda_{0}}{2}\|d^{k+1}+\frac{{\tilde{d}}^{k}}{\lambda_{0}}-\nabla u^{k+1}+\omega {\|}_{2}^{2}+\frac{\lambda_{1}}{2}\|s^{k+1}+\frac{{\tilde{s}}^{k}}{\lambda_{1}}-\varepsilon (\omega ){\|}_{2}^{2}.$$ (18)

The optimality conditions in the above case are

$$\left\{ \begin{array}{l} \;\lambda_{1}\left(\nabla_{x}^{T}\left(\nabla_{x}\omega_{x}-s_{x}^{k+1}-\frac{{\tilde{s}}_{x}^{k}}{\lambda_{1}}\right)+\nabla_{y}^{T}\left(\frac{1}{2}(\nabla_{y}\omega_{x}+\nabla_{x}\omega_{y})-s_{z}^{k+1}-\frac{{\tilde{s}}_{z}^{k}}{\lambda_{1}}\right)\right)\\ \;+\lambda_{0}\left(\omega_{x}+d_{x}^{k+1}+\frac{{\tilde{d}}_{x}^{k}}{\lambda_{0}}-\nabla_{x}u^{k+1}\right)=0,\\ \;\lambda_{1}\left(\nabla_{y}^{T}\left(\nabla_{y}\omega_{y}-s_{y}^{k+1}-\frac{{\tilde{s}}_{y}^{k}}{\lambda_{1}}\right)+\nabla_{x}^{T}\left(\frac{1}{2}(\nabla_{x}\omega_{y}+\nabla_{y}\omega_{x})-s_{z}^{k+1}-\frac{{\tilde{s}}_{z}^{k}}{\lambda_{1}}\right)\right)\\ \;+\lambda_{0}\left(\omega_{y}+d_{y}^{k+1}+\frac{{\tilde{d}}_{y}^{k}}{\lambda_{0}}-\nabla_{y}u^{k+1}\right)=0. \end{array}\right.$$ (19)

The exact minimizer of Eq (18) is formulated as

$$\begin{array}{l} \omega_{x}{}^{k+1}={\left(\lambda_{0}I+\lambda_{1}\nabla_{x}^{T}\nabla_{x}+\frac{\lambda_{1}}{2}\nabla_{y}^{T}\nabla_{y}\right)}^{+}\\ \;\left(\lambda_{0}(\nabla_{x}u^{k+1}-d_{x}^{k+1}-\frac{{\tilde{d}}_{x}^{k}}{\lambda_{0}})+\lambda_{1}\nabla_{x}^{T}(s_{x}^{k+1}+\frac{{\tilde{s}}_{x}^{k}}{\lambda_{1}})+\lambda_{1}\nabla_{y}^{T}(s_{z}^{k+1}+\frac{{\tilde{s}}_{z}^{k}}{\lambda_{1}}-\frac{\nabla_{x}\omega_{y}^{k}}{2})\right),. \end{array}$$ (20)

$$\begin{array}{l} \omega_{y}{}^{k+1}={\left(\lambda_{0}I+\lambda_{1}\nabla_{y}^{T}\nabla_{y}+\frac{\lambda_{1}}{2}\nabla_{x}^{T}\nabla_{x}\right)}^{+}\\ \;\left(\lambda_{0}(\nabla_{y}u^{k+1}-d_{y}^{k+1}-\frac{{\tilde{d}}_{y}^{k}}{\lambda_{0}})+\lambda_{1}\nabla_{y}^{T}(s_{y}^{k+1}+\frac{{\tilde{s}}_{y}^{k}}{\lambda_{1}})+\lambda_{1}\nabla_{x}^{T}(s_{z}^{k+1}+\frac{{\tilde{s}}_{z}^{k}}{\lambda_{1}}-\frac{\nabla_{y}\omega_{x}^{k+1}}{2})\right). \end{array}$$ (21)

v. Finally, the Lagrange multipliers are updated as follows:

$$\begin{array}{l} {\tilde{d}}^{k+1}={\tilde{d}}^{k}+\lambda_{0}(d^{k+1}-\nabla u^{k+1}+\omega^{k+1}),\\ {\tilde{s}}^{k+1}={\tilde{s}}^{k}+\lambda_{1}(s^{k+1}-\varepsilon (\omega^{k+1})),\\ {\tilde{r}}^{k+1}={\tilde{r}}^{k}+\mu (\sigma^{k+1}+b-Au^{k+1}). \end{array}$$ (22)

### Efficient Fourier-based solvers for subminimization problems

The pseudoinverse is used to solve the subminimization problem w.r.t *u* (Eq (16)) in the reconstruction algorithm. This solution may only work for a toy example but is far less feasible for practical CT reconstruction because CT data are excessively large. This subproblem is conventionally solved using iterative methods, such as conjugate gradient [38–39] and nonmonotone alternating direction algorithm [40], which may also lead to significant computation and memory consumption.

Our observation shows that an exact solution to the *u* -subproblem is generally unnecessary. Rather, an approximate solution can be used. We introduce a proximal point technique [41,42] to avoid the prohibitive cost and solve the subproblem efficiently.

In Eq (14), $\frac{1}{2}\|Au-b-\sigma^{k}{\|}_{2}^{2}$ can be linearized at current point *u*^*k*^ in each iteration:

$$\frac{1}{2}\|Au-b-\sigma^{k}{\|}_{2}^{2}\approx \frac{1}{2}\|Au^{k}-b-\sigma^{k}{\|}_{2}^{2}+\rho^{T}\left(u-u^{k}\right)+\frac{1}{2\tau }\|u-u^{k}{\|}_{2}^{2},$$ (23)

where *ρ* = A^*T*^(*Au*−*b*−*σ*^*k*^) denotes the gradient at *u*^*k*^, and *τ*>0 is a parameter.

Then, the *u* -subproblem can be transformed to an approximation problem by adding the proximal term

$$\begin{array}{l} \underset{u}{\text{min}}\frac{\mu }{2}\left(\|Au^{k}-b-\sigma^{k}{\|}_{2}^{2}+2\rho^{T}\left(u-u^{k}\right)+\frac{1}{\tau }\|u-u^{k}{\|}_{2}^{2}\right)\\ \;-{\tilde{r}}^{T}(Au-b-\sigma^{k})+\frac{\lambda_{0}}{2}\|d^{k+1}+\frac{{\tilde{d}}^{k}}{\lambda_{0}}-\nabla u+\omega^{k}{\|}_{2}^{2}. \end{array}$$ (24)

The first-order necessary conditions for optimality are

$$\left(\frac{\mu }{\tau }I+\lambda_{0}\nabla^{T}\nabla \right)u=\frac{\mu }{\tau }u^{k}-\mu \rho +A^{T}\tilde{r}+\lambda_{0}\nabla^{T}\left(d^{k+1}+\frac{{\tilde{d}}^{k}}{\lambda_{0}}+\omega^{k}\right).$$ (25)

The circulant matrices can be diagonalized under the Fourier transform, and ▽^*T*^▽ is a constant and block-circulant matrix. Thus, under the periodic boundary condition for *u*, the coefficient matrix $\left(\frac{\mu }{\tau }I+\lambda_{0}\nabla^{T}\nabla \right)$ can be diagonalized blockwise by the 2D Fourier matrix. Denoting. $G=\text{diag}\left[F\left(\frac{\mu }{\tau }I+\lambda_{0}\nabla^{T}\nabla \right)F^{-1}\right]$., where F stands for a Fourier transform matrix implemented by 2D fast Fourier transform (FFT), diag[*M*] is a vectorization diagonal operator which returns a vector constructed by the principal diagonal entities of *M*, we have

$$u^{k+1}=F^{-1}\left(F\left(\frac{\mu }{\tau }u^{k}-\mu \rho +A^{T}\tilde{r}+\lambda_{0}\nabla^{T}\left(d^{k+1}+{\tilde{d}}^{k}/\lambda_{0}+\omega^{k}\right)\right)/G\right).$$ (26)

Consequently, the *u*-subproblem can be solved by only two FFTs, thereby avoiding the costly calculation of pseudoinverse.

We further exploit the fast calculation of the solution to the *ω*-subproblem. For convenience, Eq (19) can be reformulated by grouping like terms

$$[\begin{array}{l} C_{1}\\ C_{3} \end{array}\;\begin{array}{l} C_{3}^{T}\\ C_{2} \end{array}]\left[\begin{array}{l} \omega_{x}\\ \omega_{y} \end{array}\right]=\left[\begin{array}{l} B_{1}\\ B_{2} \end{array}\right],$$ (27)

where the coefficient matrices are listed as follows:

$$\left\{ \begin{array}{l} C_{1}=\lambda_{0}I+\lambda_{1}(\nabla_{x}^{T}\nabla_{x}+\frac{1}{2}\nabla_{y}^{T}\nabla_{y}),\\ C_{2}=\lambda_{0}I+\lambda_{1}(\nabla_{y}^{T}\nabla_{y}+\frac{1}{2}\nabla_{x}^{T}\nabla_{x}),\\ C_{3}=\frac{\lambda_{1}}{2}\nabla_{x}^{T}\nabla_{y},\\ B_{1}=\lambda_{0}\left(\nabla_{x}u^{k+1}-d_{x}^{k+1}-{\tilde{d}}_{x}^{k}/\lambda_{0}\right)+\lambda_{1}\left(\nabla_{x}^{T}(s_{x}^{k+1}+{\tilde{s}}_{x}^{k}/\lambda_{1})+\nabla_{y}^{T}(s_{z}^{k+1}+{\tilde{s}}_{z}^{k}/\lambda_{1})\right),\\ B_{2}=\lambda_{0}\left(\nabla_{y}u^{k+1}-d_{y}^{k+1}-{\tilde{d}}_{y}^{k}/\lambda_{0}\right)+\lambda_{1}\left(\nabla_{y}^{T}(s_{y}^{k+1}+{\tilde{s}}_{y}^{k}/\lambda_{1})+\nabla_{x}^{T}(s_{z}^{k+1}+{\tilde{s}}_{z}^{k}/\lambda_{1})\right). \end{array}\right.$$ (28)

Similarly, the coefficient matrix is blockwise diagonal. Multiplying a preconditioned matrix with Fourier transform converts the linear system into the following form:

$$[\begin{array}{l} F\\ 0 \end{array}\;\begin{array}{l} 0\\ F \end{array}][\begin{array}{l} C_{1}\\ C_{3} \end{array}\;\begin{array}{l} C_{3}^{T}\\ C_{2} \end{array}][\begin{array}{l} F\\ 0 \end{array}\;{\begin{array}{l} 0\\ F \end{array}]}^{-1}\left[\begin{array}{l} F\omega_{x}\\ F\omega_{y} \end{array}\right]=[\begin{array}{l} F\\ 0 \end{array}\;\begin{array}{l} 0\\ F \end{array}]\left[\begin{array}{l} B_{1}\\ B_{2} \end{array}\right].$$ (29)

Let $\hat{C_{i}}=\text{diag[}F\;C_{i}\;F^{-1}\text{]}$, we have

$$\left\{ \begin{array}{l} \hat{C_{1}}.*(F\omega_{x})+\hat{C_{3}^{T}}.*(F\omega_{y})=FB_{1}\\ \hat{C_{3}}.*(F\omega_{x})+\hat{C_{2}}.*(F\omega_{y})=FB_{2} \end{array}\right.,$$ (30)

where.* is componentwise multiplication.

Then, we can obtain the following closed forms:

$$\left\{ \begin{array}{l} \omega_{x}{}^{k+1}=F^{-1}\left({\left|\begin{array}{l} FB_{1}\;\hat{C_{3}^{T}}\\ FB_{2}\;\hat{C_{2}} \end{array}\right|}_{*}./{\left|\begin{array}{l} \hat{C_{1}}\;\hat{C_{3}^{T}}\\ \hat{C_{3}}\;\hat{C_{2}} \end{array}\right|}_{*}\right)\\ \omega_{y}{}^{k+1}=F^{-1}\left({\left|\begin{array}{l} \hat{C_{1}}\;FB_{1}\\ \hat{C_{3}}\;FB_{2} \end{array}\right|}_{*}./{\left|\begin{array}{l} \hat{C_{1}}\;\hat{C_{3}^{T}}\\ \hat{C_{3}}\;\hat{C_{2}} \end{array}\right|}_{*}\right) \end{array}\right.,$$ (31)

where |·|_*_ is defined as

$${\left|\begin{array}{l} M_{11}\;M_{12}\\ M_{21}\;M_{22} \end{array}\right|}_{*}=M_{11}.*M_{22}-M_{12}.*M_{21}.$$ (32)

The augmented Lagrangian function (8) is expected to be minimized by solving the four subproblems alternately. All of the subproblems in the proposed algorithm have noticeably efficient solutions: the *d*-subproblem and the *s*-subproblem are solved by using easy-to-compute p-shrinkage operators; the *u*-subproblem and the *ω*-subproblem are converted to Fourier-based formulations, which can be rapidly calculated using FFT techniques. Thus, the proposed algorithm is efficient and practical for the low cost in each iteration.

### Pseudocode of the TGpV-ADM reconstruction algorithm

In summary, the workflow of present TGpV-ADM method for X-ray CT image reconstruction is listed in Algorithm 1.

**Algorithm 1. Constrained TGpV minimization in the ADM (TGpV-ADM) framework.**

Input *A*,*b*,*μ*,*λ*_0_,*λ*_1_,*α*_0_,*α*_1_,*τ*,*e*, initialize *d*^0^,*s*^0^,*u*^0^,*ω*^0^, and *k* = 0. Given *p* ∈(0, 1):

**While** “not converged,” **Do**

  (1) Update *d*^*k*+1^ by $d^{k+1}\leftarrow shrink_{p}(\nabla u^{k}-\omega^{k}-\frac{{\tilde{d}}^{k}}{\lambda_{0}},\alpha_{0}/\lambda_{0}),$

  (2) Update *s*^*k*+1^ by $s^{k+1}\leftarrow shrink_{p}(\varepsilon (\omega^{k})-\frac{{\tilde{s}}^{k}}{\lambda_{1}},\alpha_{1}/\lambda_{1}),$

  (3) Update *u*^*k*+1^ by $u^{k+1}\leftarrow F^{-1}\left(F\left(\frac{\mu }{\tau }u^{k}-\mu \rho +A^{T}\tilde{r}+\lambda_{0}\nabla^{T}\left(d^{k+1}+{\tilde{d}}^{k}/\lambda_{0}+\omega^{k}\right)\right)/G\right),$

  (4) Update *σ*^*k*+1^ by $\sigma^{k+1}\leftarrow \text{min}\left\{1,\;e/{\left\|Au^{k+1}-b\right\|}_{2}^{}\right\}\cdot \left(Au^{k+1}-b\right),$

  (5) Update *ω*^*k*+1^ by $\left\{ \begin{array}{l} \omega_{x}{}^{k+1}\leftarrow F^{-1}\left({\left|\begin{array}{l} FB_{1}\;\hat{C_{3}^{T}}\\ FB_{2}\;\hat{C_{2}} \end{array}\right|}_{*}./{\left|\begin{array}{l} \hat{C_{1}}\;\hat{C_{3}^{T}}\\ \hat{C_{3}}\;\hat{C_{2}} \end{array}\right|}_{*}\right)\\ \omega_{y}{}^{k+1}\leftarrow F^{-1}\left({\left|\begin{array}{l} \hat{C_{1}}\;FB_{1}\\ \hat{C_{3}}\;FB_{2} \end{array}\right|}_{*}./{\left|\begin{array}{l} \hat{C_{1}}\;\hat{C_{3}^{T}}\\ \hat{C_{3}}\;\hat{C_{2}} \end{array}\right|}_{*}\right) \end{array}\right.,$

  (6) Update multipliers by $\left\{ \begin{array}{l} {\tilde{d}}^{k+1}\leftarrow {\tilde{d}}^{k}+\lambda_{0}(d^{k+1}-\nabla u^{k+1}+\omega^{k+1})\\ {\tilde{s}}^{k+1}\leftarrow {\tilde{s}}^{k}+\lambda_{1}(s^{k+1}-\varepsilon (\omega^{k+1}))\\ {\tilde{r}}^{k+1}\leftarrow {\tilde{r}}^{k}+\mu (b+\sigma^{k+1}-Au^{k+1}) \end{array}\right.,$

  (7) *k* ← *k* + 1.

**End Do**

Obtain reconstruction result:*u*.

### Parameter selections

Parameters *μ*, *λ*_0_, and *λ*_1_ are used to balance the data fidelity and two regularization terms. To get the optimal performance, the values of them should be set in accordance with both the noise level in the observation and the sparsity level of the underlying image. Generally, the higher the noise level is, the smaller *μ* should be. Actually, they are often empirically selected by visual inspection. Based on our experience, a simple way to choose them is to try different values from 2^3^ up to 2^13^ and compare the recovered images. For most CT imaging cases, the value of parameter *μ* could be given larger than that of *λ*_0_ and *λ*_1_.

The positive weights *α*_0_ and *α*_1_ are used to balance the first and second derivatives. Proper values of them should be chosen based on sparsity feature of the underlying image. Generally, *α*_0_, *α*_1_∈[1, 4] is suitable for most applications.

Parameter *p* is in (0, 1) and plays a vital role. The penalty function is somewhat approximated but not strictly equivalent to *l*_p_ minimization; thus, a considerable value should be determined rigorously. Based on our experience, *p*∈[0.5, 0.9] is adequate for noiseless datasets, and *p*∈[0.7, 0.9] is adequate for noisy datasets.

### Performance evaluations

For the quantitative evaluation of the TGpV-ADM algorithm, the root-mean-square error (RMSE), peak signal-to-noise ratio (PSNR), and normalized root mean square distance (NRMSD) [43] are used as measures of the reconstruction quality. The RMSE, PSNR and NRMSD are defined as follows:

$$RMSE\;=\;\sqrt{\frac{\sum_{i=1}^{N}{\left|f_{i}-u_{i}\right|}^{2}}{N}},$$ (33)

$$PSNR\;=10{\text{log}}_{10}\left(\frac{MAX^{2}\left(f\right)}{\frac{1}{N}\sum_{i=1}^{N}{\left|f_{i}-u_{i}\right|}^{2}}\right)\;$$ (34)

$$NRMSD\;=\;\sqrt{\frac{\sum_{i=1}^{N}{\left|f_{i}-u_{i}\right|}^{2}}{\sum_{i=1}^{N}f_{i}{}^{2}}},$$ (35)

where *f* and *u* denote the ideal phantom and the reconstruction, respectively, and *i* indexes the pixels in the image. *N* is the total number of pixels in the image. An RMSE/NRMSD value close to zero suggests high similarity to the ideal phantom image. And a higher PSNR indicates that the image is of higher quality.

## Results

To validate and evaluate the performance of the proposed method, three types of projection data (computer-simulated digital Moby phantom, computer-simulated digital CS-phantom, and experimental anthropomorphic phantom projection data) were used in the experiments. Both computer-simulated digital phantom projection data and real CT projection data were used to compare the proposed TGpV-ADM method with the standard TV-ADM [42], TpV-ADM [35], and TGV-ADM [27] algorithms. The implementations of these three comparison methods are described in S1 Appendix. All of the experiments were performed under Matlab 2012a running on an HP-Z820 workstation with Intel Xeon E5-2650 dual-core CPU 2.60 GHz.

To assess the quantitative evaluations of image quality (RMSE, PSNR, NRMSD), the tests of statistical significance were carried out using 60 phases of the Moby phantom. First, we performed the F-test. If the p-value of F-test was larger than 0.05, the t-test was then carried out; If the p-value was less than 0.05, the variances of the two samples could not be assumed to be equal and the Welch’s t-test [44] was then carried out. In the statistical significance tests, variable were expressed as Mean ± standard deviations.

### Digital Moby phantom study

**Fig 1:**
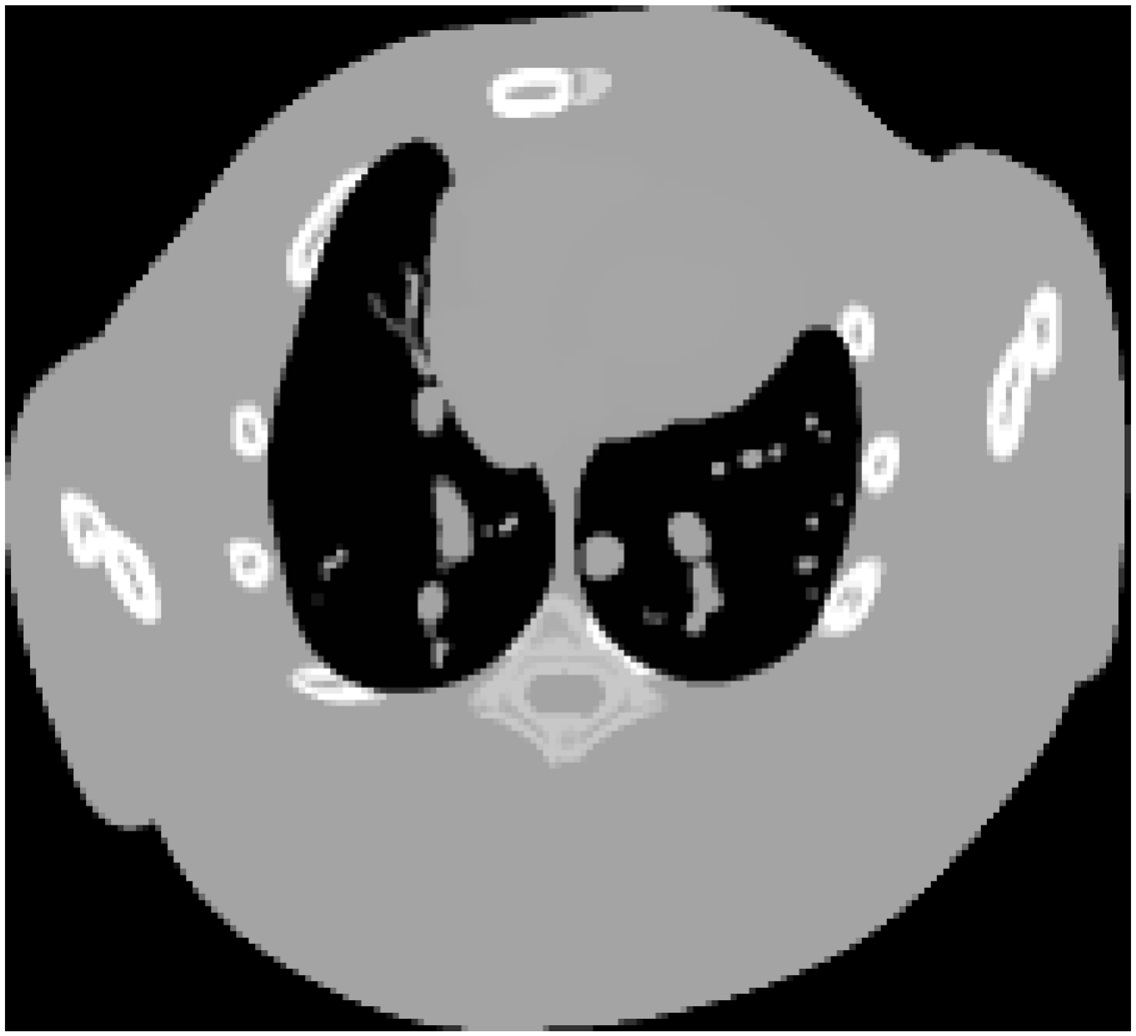
Digital Moby phantom used in simulation study. Display window is [0.2, 1.0].

In the first group of simulation study, a digital Moby phantom [45–46] was used to simulate the few-view projection data. The Moby phantom, which modeled a 3D mouse anatomy, was often used in simulation studies for single photon emission computed tomography and X-ray CT. One typical frame of the phantom is shown in Fig 1 (or S1 Fig).

### Noise-free cases

For the CT projection simulation, we chose a geometry that was representative of a fan-beam CT scanner setup. The imaging configurations were as follows: (1) the projection data comprised 36 projections at an interval of 5° onto a 720-bin linear detector array, (2) the distance from the detector to the X-ray source was 600 mm, (3) the distance from the rotation center to the source was 300 mm, and (4) the space of each detector bin was 0.1 mm. All of the reconstructed images comprised a 256 × 256 square of pixels. The size of each pixel was 0.1 mm × 0.1 mm. Each projection datum along an X-ray through the sectional image was calculated as the intersection length of an X-ray with a pixel.

For four ADM-based algorithms, the parameters of ADM framework were the same in the experiments:*μ* and *λ*_0_ were set to 256 and 64, respectively; *τ* was set to 1.3. As the image is piecewise constant in most areas, for the TGV-ADM and TGpV-ADM methods, *α*_0_, *α*_1_, and *λ*_1_ were set to 1, 2, and 64, respectively. For the TpV-ADM and TGpV-ADM algorithms, *p* was set to 0.9. Considering the absence of noise from the projection data, we set *e* to 0. The number of iterations for each reconstruction was 600.

**Fig 2:**
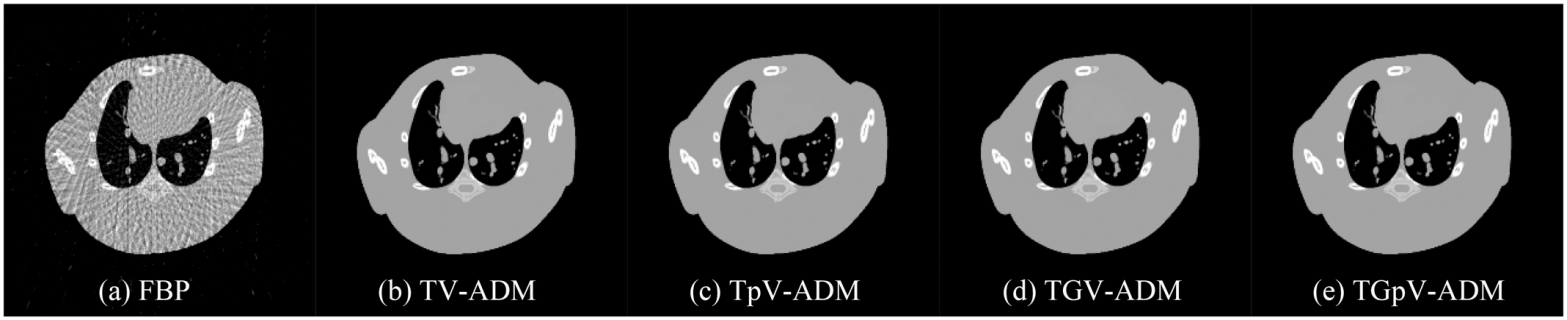
Image reconstruction of the Moby phantom from noise-free projection dataset. Results of the (a) FBP, (b) TV-ADM, (c) TpV-ADM, (d) TGV-ADM, and (e) TGpV-ADM minimization methods. Display window is [0.2, 1.0].

**Fig 3:**
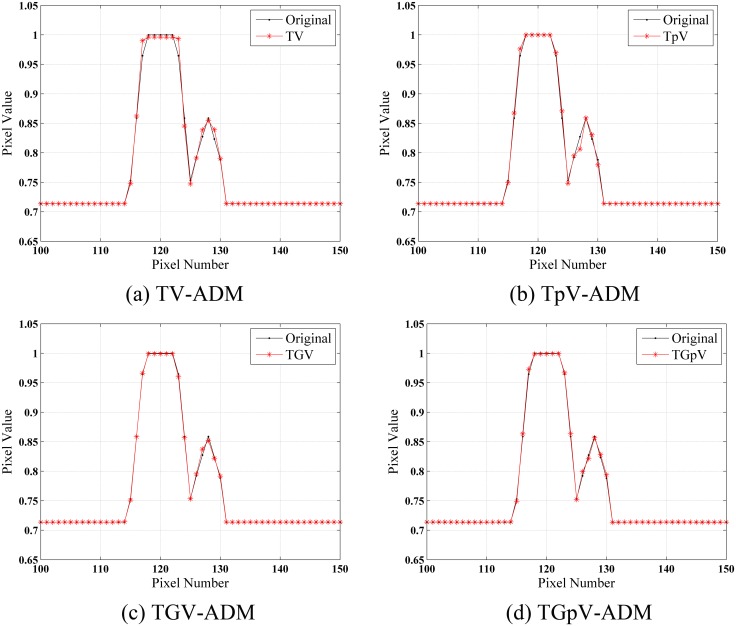
Horizontal profiles (52th row) in the reconstruction results of the Moby phantom from noise-free projection dataset. Results of the (a) TV-ADM, (b) TpV-ADM, (c) TGV-ADM, and (d) TGpV-ADM minimization methods.

The images reconstructed from the four methods in the noise-free cases are shown in Fig 2. All methods could recover image well from sparse projections in visual inspection. To visualize the difference in detail, horizontal profiles of the resulting images (Fig 3) are drawn across the 52th row, that is, from the 100th column to the 150th column. As one can see, the images obtained by use of the TV-ADM and TpV-ADM algorithms are reasonably accurate with only small distortions, and the TGpV-ADM method can produce more closely matching results.

Table 1 lists the RMSE, PSNR, and NRMSD measures of the images reconstructed by different algorithms with 600 iterations. From Table 1, it finds that the TGpV-ADM algorithm also outperforms other counterparts when using objective evaluation metrics.

**Table 1:** Evaluations of the results reconstructed by different methods from noise-free projection dataset in digital Moby phantom studies.

	RMSE	PSNR	NRMSD
TV-ADM	2.7626e-03	51.1737	7.0661e-03
TpV-ADM	1.6702e-03	55.5448	4.2719e-03
TGV-ADM	1.2492e-03	58.0675	3.1951e-03
TGpV-ADM	9.9026e-04	60.0850	2.5329e-03

### Noisy cases

To check the capability of the proposed algorithm further, we carried out the experiments to reconstruct images from noisy projections. Noise is generated using a Poisson model [47]:

$$\bar{p}\left(k,\lambda \right)=\frac{\lambda^{k}}{k!}e^{-\lambda },\;\;\;\lambda =N_{0}\text{exp}(-p),$$ (36)

where *N*_*0*_ is the incident X-ray intensity, *p* denotes the normalized projections in real space, and $\bar{p}$ denotes the normalized projections with added Poisson noise. *k* indexes the pixels in the projection data.

Admissible reconstruction needs more projections than noiseless cases because of inconsistencies in the data. Thus, the imaging configuration was the same with the noise-free group except the projection acquisition. The total view number of the experiment was 90.

In this section, three cases with different noisy levels were considered. The initial numbers of photons *N*_*0*_ were set to 5×10^6^, 2×10^6^, and 5×10^5^ for noisy case 1, 2, and 3, respectively. For noisy case 1 and 2, the parameter *μ* of ADM framework in the four algorithms was set to 64, and for noisy case 3, *μ* was set to 32. The parameter *λ*_0_ was set to 16. *τ* was set to 1.3. For the TGV-ADM and TGpV-ADM methods, *α*_0_, α_1_, and *λ*_1_ were set to 1, 2, and 16, respectively. For the TpV-ADM and TGpV-ADM algorithms, *p* was set to 0.9. As the noise levels of images in three cases were different, the numbers of iterations in three cases were set to 200, 180, and 160, respectively.

**Fig 4:**
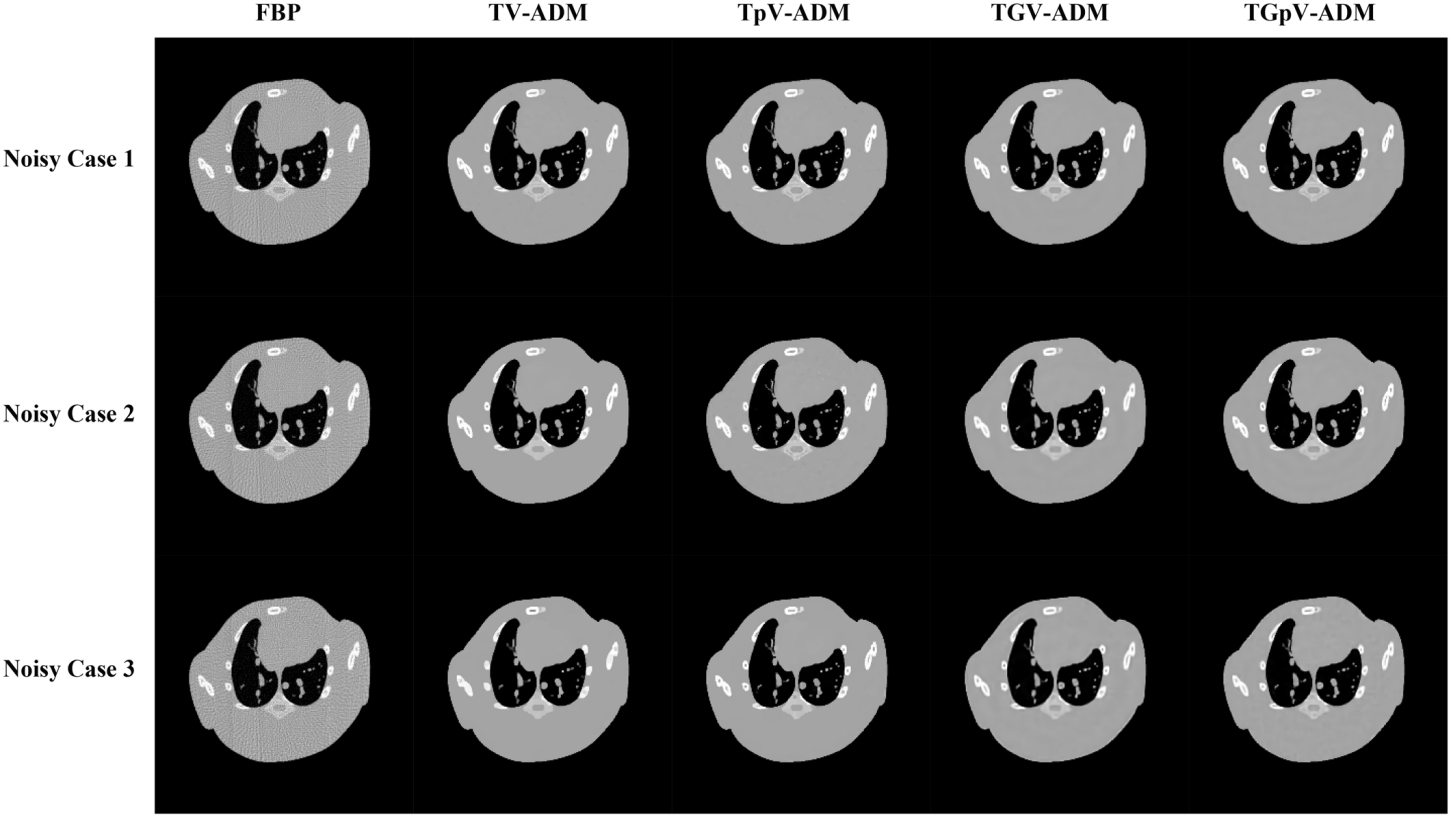
Image reconstruction of the Moby phantom from noisy projection dataset. Rows from the top to the bottom are the reconstructed results from three groups of projections with different noise levels. The photon number N_0_ of noisy case 1, 2, 3 are 5×10^6^, 2×10^6^, and 5×10^5^, respectively. From left to right in each row, results of the FBP, TV-ADM, TpV-ADM, TGV-ADM, and TGpV-ADM methods are presented.

**Fig 5:**
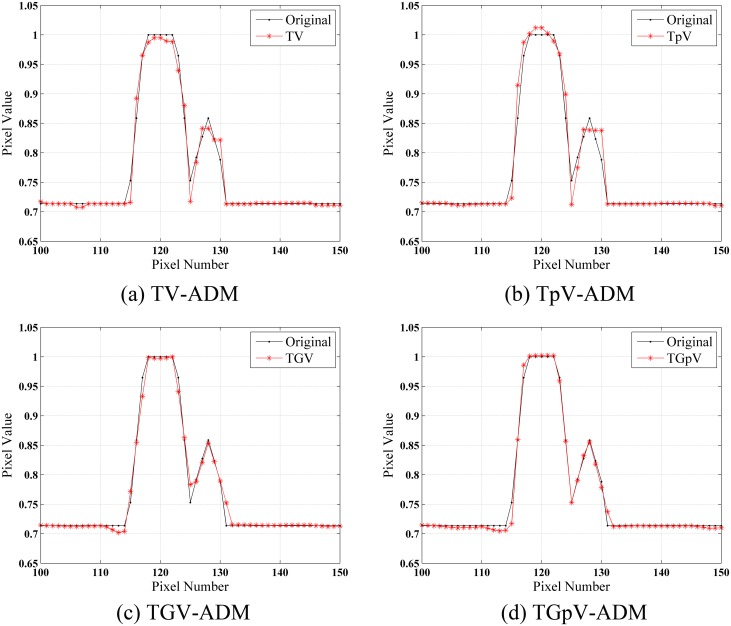
Horizontal profiles (52th row) in the reconstruction results of the Moby phantom from the first group of noisy projection dataset (N_0_ = 5×10^6^). Results of the (a) TV-ADM, (b) TpV-ADM, (c) TGV-ADM, and (d) TGpV-ADM minimization methods.

**Fig 6:**
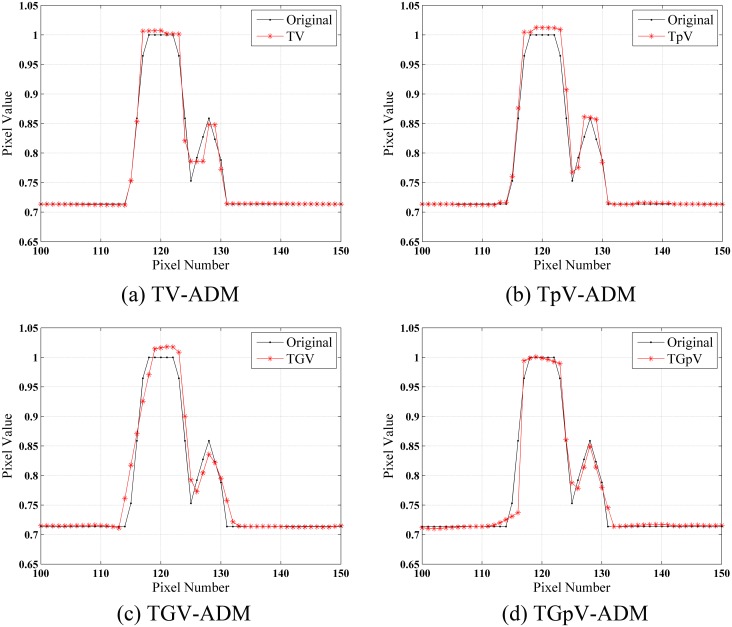
Horizontal profiles (52th row) in the reconstruction results of the Moby phantom from the second group of noisy projection dataset (N_0_ = 2×10^6^). Results of the (a) TV-ADM, (b) TpV-ADM, (c) TGV-ADM, and (d) TGpV-ADM minimization methods.

**Fig 7:**
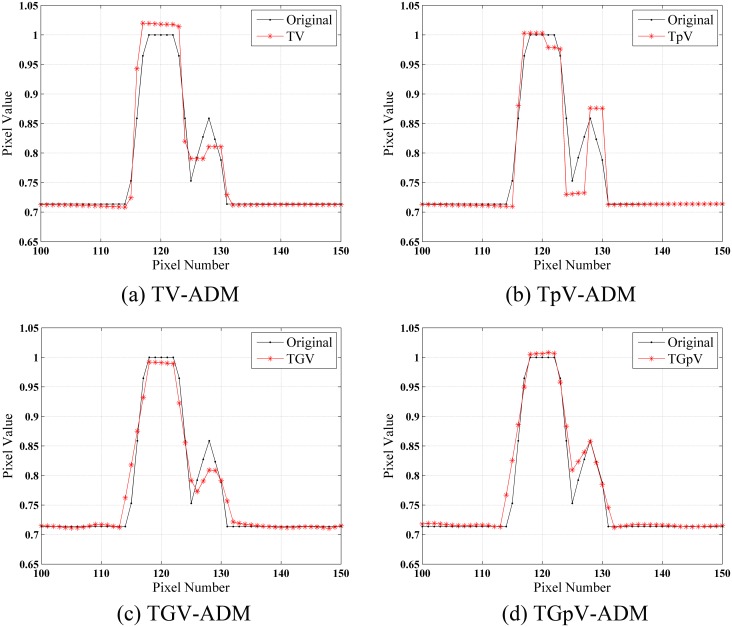
Horizontal profiles (52th row) in the reconstruction results of the Moby phantom from the third group of noisy projection dataset (N_0_ = 5×10^5^). Results of the (a) TV-ADM, (b) TpV-ADM, (c) TGV-ADM, and (d) TGpV-ADM minimization methods.

The images reconstructed by FBP, TV-ADM, TpV-ADM, TGV-ADM, and TGpV-ADM methods from three different groups of noisy projections are shown in Fig 4. The profiles of these images along the 52th horizontal rows of three different noisy cases are indicated in Figs 5, 6 and 7, respectively. The profiles show that the TGV-ADM and TGpV-ADM reconstructions contain a little deviation from the original phantom and the TV-ADM and TpV-ADM reconstruction have some distortions which are evident in the shown profile plots. Interestingly, the gains from the TGpV-ADM method are more noticeable compared with those from the TGV-ADM method.

The RMSE, PSNR and NRMSD of the reconstructions from the different methods was calculated, and the calculation results are listed in Table 2. The quantitative results from the proposed TGpV-ADM algorithm showed better results than that from other algorithms in terms of the three measures, which agrees with the findings in Table 1.

**Table 2:** Evaluations of the results reconstructed by different methods from noisy projection dataset in digital Moby phantom studies.

	Noisy Case 1			Noisy Case 2			Noisy Case 3		
	RMSE	PSNR	NRMSD	RMSE	PSNR	NRMSD	RMSE	PSNR	NRMSD
TV-ADM	8.0938e-03	41.8370	2.0702e-02	9.8109e-03	40.1658	2.5094e-02	1.2874e-02	37.8058	3.2929e-02
TpV-ADM	6.7564e-03	43.4057	1.7281e-02	8.7009e-03	41.2087	2.2255e-02	1.2572e-02	38.0118	3.2157e-02
TGV-ADM	6.3849e-03	43.8969	1.6331e-02	8.9127e-03	40.9998	2.2797e-02	1.2617e-02	37.9808	3.2272e-02
TGpV-ADM	5.9849e-03	44.4589	1.5308e-02	8.9127e-03	42.1914	1.9874e-02	1.1918e-02	38.4758	3.0484e-02

To further assess the performance evaluations of image quality reconstructed by different algorithms, we performed the tests of statistical significance using 60 phases of the Moby phantom. The statistical mean values of performance evaluations of the images reconstructed by different algorithms with 600 iterations from noise-free projections are summarized in Table 3. The corresponding F-test and t-test analysis results are summarized in Table 4. Similarly, for the three groups of experiments that the images reconstructed from noisy projections, the statistical mean values of performance evaluations are summarized in Tables 5, 6 and 7, respectively. The corresponding F-test and t-test analysis results are summarized in Tables 8, 9 and 10, respectively.

**Table 3:** Summary of statistical analysis results of performance evaluations of the images reconstructed by different methods from noise-free projection dataset in digital Moby phantom studies.

	TV-ADM(A)	TpV-ADM(B)	TGV-ADM(C)	TGpV-ADM(D)
RMSE	0.002635±0.0001233	0.001660±0.00009580	0.001437±0.0006592	0.001008±0.0001160
PSNR	51.5836±0.4064	55.5951±0.5002	56.8491±2.8072	59.9290±0.8252
NRMSD	0.006679±0.0003628	0.004215±0.0002162	0.003599±0.001689	0.002507±0.0002994

**Table 4:** Summary of F-test and t-test analysis results of performance evaluations of image quality between different algorithms in noise-free case in digital Moby phantom studies.

	p_F_-value			p-value		
	A vs. D	B vs. D	C vs. D	A vs. D	B vs. D	C vs. D
RMSE	0.639	0.145	<0.001	<0.0001	<0.0001	<0.0001
PSNR	<0.001	<0.001	<0.001	<0.0001	<0.0001	<0.0001
NRMSD	0.143	0.014	<0.001	<0.0001	<0.0001	<0.0001

**Table 5:** Summary of statistical analysis results of performance evaluations of the images reconstructed by different methods in noisy case 1 (N_0_ = 5×10^6^) for 60 phases of the Moby phantom.

	TV-ADM(A)	TpV-ADM(B)	TGV-ADM(C)	TGpV-ADM(D)
RMSE	0.007859±0.0006082	0.006772±0.0003184	0.006354±0.0003444	0.005946±0.0003335
PSNR	42.0922±0.6599	43.3856±0.4083	43.9396±0.4773	44.5155±0.4913
NRMSD	0.01969±0.001544	0.01691±0.0008381	0.01572±0.0009133	0.01497±0.0008090

**Table 6:** Summary of statistical analysis results of performance evaluations of the images reconstructed by different methods in noisy case 2 (N_0_ = 2×10^6^) for 60 phases of the Moby phantom.

	TV-ADM(A)	TpV-ADM(B)	TGV-ADM(C)	TGpV-ADM(D)
RMSE	0.009484±0.0006237	0.008490±0.0003828	0.008340±0.0003960	0.007749±0.0004074
PSNR	40.4603±0.5687	41.4216±0.3912	41.5769±0.4101	42.2145±0.4597
NRMSD	0.02386±0.001628	0.02118±0.001036	0.02103±0.001095	0.01939±0.0009966

**Table 7:** Summary of statistical analysis results of performance evaluations of the images reconstructed by different methods in noisy case 3 (N_0_ = 5×10^5^) for 60 phases of the Moby phantom.

	TV-ADM(A)	TpV-ADM(B)	TGV-ADM(C)	TGpV-ADM(D)
RMSE	0.01251±0.0009569	0.01191±0.0008555	0.01244±0.0005268	0.01168±0.0005963
PSNR	38.0536±0.6548	38.4822±0.6115	38.1025±0.3675	38.6497±0.4514
NRMSD	0.03154±0.002434	0.03012±0.002141	0.03140±0.001540	0.02919±0.001592

**Table 8:** Summary of F-test and t-test analysis results of performance evaluations of image quality between different algorithms in noisy case 1 (N_0_ = 5×10^6^) for 60 phases of the Moby phantom.

	p_F_-value			p-value		
	A vs. D	B vs. D	C vs. D	A vs. D	B vs. D	C vs. D
RMSE	<0.001	0.723	0.806	<0.0001	<0.0001	<0.0001
PSNR	<0.001	0.158	0.825	<0.0001	<0.0001	<0.0001
NRMSD	<0.001	0.787	0.354	<0.0001	<0.0001	<0.0001

**Table 9:** Summary of F-test and t-test analysis results of performance evaluations of image quality between different algorithms in noisy case 2 (N_0_ = 2×10^6^) for 60 phases of the Moby phantom.

	p_F_-value			p-value		
	A vs. D	B vs. D	C vs. D	A vs. D	B vs. D	C vs. D
RMSE	0.001	0.635	0.828	<0.0001	<0.0001	<0.0001
PSNR	0.105	0.218	0.383	<0.0001	<0.0001	<0.0001
NRMSD	<0.001	0.769	0.472	<0.0001	<0.0001	<0.0001

**Table 10:** Summary of F-test and t-test analysis results of performance evaluations of image quality between different algorithms in noisy case 3 (N_0_ = 5×10^5^) for 60 phases of the Moby phantom.

	p_F_-value			p-value		
	A vs. D	B vs. D	C vs. D	A vs. D	B vs. D	C vs. D
RMSE	<0.001	0.006	0.344	<0.0001	0.0006	<0.0001
PSNR	0.005	0.021	0.117	<0.0001	0.0008	<0.0001
NRMSD	0.001	0.024	0.800	<0.0001	0.0008	<0.0001

In the noise-free case, noisy case 1 and noisy case 2, there are significant differences in the values of RMSE, PSNR, and NRMSD between other algorithms and TGpV-ADM algorithm (p<0.0001). The values of RMSE and NRMSD by TGpV-ADM algorithm are significantly lower than that of other three algorithms. Meanwhile, the values of PSNR by TGpV-ADM algorithm is higher than that of TV-ADM, TpV-ADM, and TGV-ADM algorithms. In the noisy case 3, there are significant differences in the values of RMSE, PSNR, and NRMSD between TV-ADM, TGV-ADM algorithms and TGpV-ADM algorithm (p<0.0001). There are obvious statistical differences between TGpV-ADM and TpV-ADM in RMSE (p = 0.0006<0.05), PSNR (P = 0.0008<0.05), NRMSD (P = 0.0008<0.05).

The average computation time of TV-ADM, TpV-ADM, TGV-ADM, and TGpV-ADM methods are listed in Table 11. The average computation time of TGV-ADM, TGpV-ADM are longer than TV-ADM and TpV-ADM, which are due to the extra computation that takes by the subminimization problem of second derivatives. Compared with original TGV-ADM method, TGpV-ADM requires only a small increase in time computation.

**Table 11:** Running time (in CPU seconds) of different methods in digital Moby phantom studies.

	TV-ADM	TpV-ADM	TGV-ADM	TGpV-ADM
Noise-free Cases	38.285	41.418	48.136	51.462
Noisy Case 1	22.336	23.864	26.032	28.105
Noisy Case 2	19.766	20.916	22.126	23.621
Noisy Case 3	16.882	17.452	18.860	19.692

### Digital CS-phantom study

**Fig 8:**
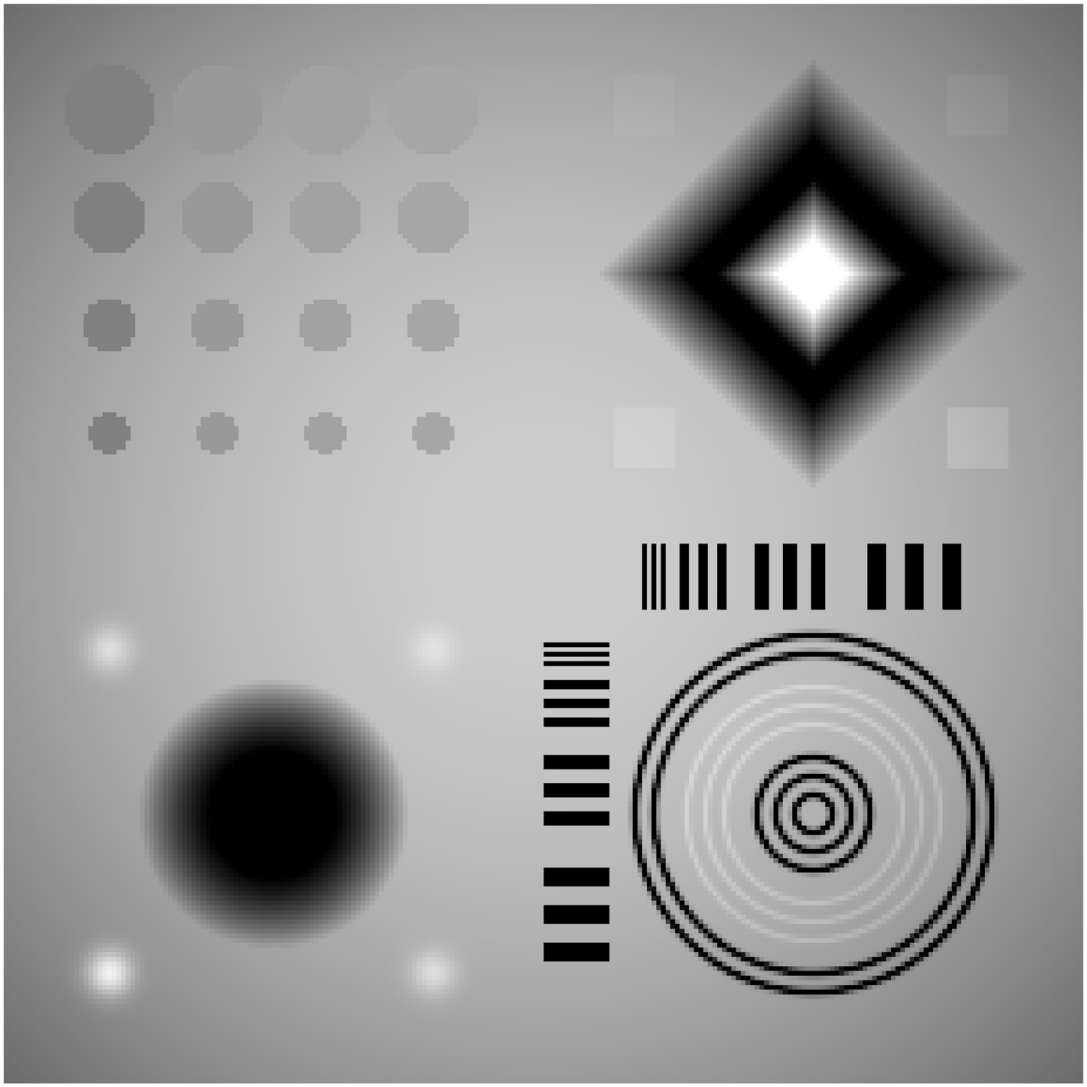
Digital CS-phantom used in simulation study. Display window is [0.1, 0.6].

To demonstrate and validate our new method for the objects with piecewise polynomial feature, a digital CS-phantom [48–49] designed for compressed sensing MRI reconstruction was used to simulate the few-view projection data. The phantom image is composed of four quadrants: Quadrant I contains a large diagonal ramp and low-contrast squares; Quadrant II contains 16 low-contrast circles; Quadrant III contains a large quadratic hole and four Gaussian bumps; and Quadrant IV contains line pairs and concentric circles with a range of spacing. This phantom is not sparse under a gradient transformation and could provide features amenable for real anatomical studying. For reference, the typical CS-phantom is shown in Fig 8 (or S2 Fig).

### Noise-free cases

In this simulation, the imaging configurations were same with the noise-free group in digital Moby phantom studies. For four ADM-based algorithms, the parameters of ADM framework were the same in the experiments: *μ* and *λ*_0_ were set to 512 and 64, respectively; *τ* was set to 1.3. For the TGV-ADM and TGpV-ADM methods, *α*_0_, *α*_1_ and *λ*_1_ were set to 1, 1, and 64, respectively. For the TpV-ADM and TGpV-ADM algorithms, *p* was set to 0.7. Considering the absence of noise from the projection data, we set *e* to 0. The number of iterations for each reconstruction was 800.

**Fig 9:**
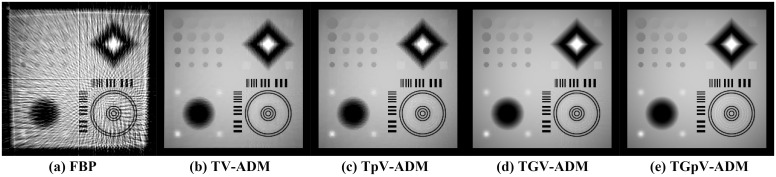
Image reconstruction of the CS-phantom from noise-free projection dataset. Results of the (a) FBP, (b)TV-ADM, (c) TpV-ADM, (d) TGV-ADM, and (e) TGpV-ADM minimization methods. Display window is [0.1, 0.6].

**Fig 10:**
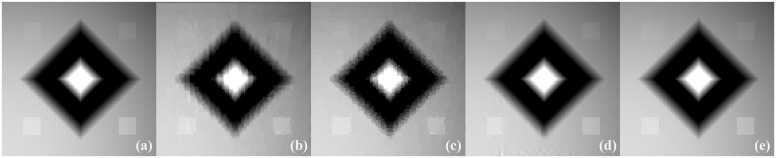
Reconstructed ROIs of Quadrant I from noise-free projection dataset. From left to right are (a) original image, results of the (b) TV-ADM, (c) TpV-ADM, (d) TGV-ADM, and (e) TGpV-ADM minimization methods. Display window is [0.2, 0.55].

**Fig 11:**
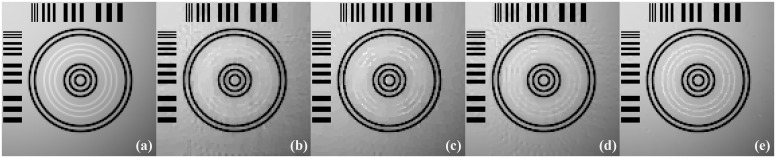
Reconstructed ROIs of Quadrant IV from noise-free projection dataset. From left to right are (a) original image, results of the (b) TV-ADM, (c) TpV-ADM, (d) TGV-ADM, and (e) TGpV-ADM minimization methods. Display window is [0.2, 0.55].

The images reconstructed from the four methods in the noise-free cases are shown in Fig 9. To reveal texture details, the zoomed region of interest (ROI) images of Quadrant I and Quadrant IV are shown in Figs 10 and 11, respectively. TV-ADM and TpV-ADM reconstructions have many blocky artifacts in smoothly varying image regions, especially in the region of diagonal ramp in Fig 10. The TGV-ADM and TGpV-ADM methods efficiently avoid the staircase effect. Compared with the TV-ADM, TpV-ADM, and TGV-ADM methods, the TGpV-ADM method can obtain more accurate images and show better recovery of details and subtle lesions.

**Fig 12:**
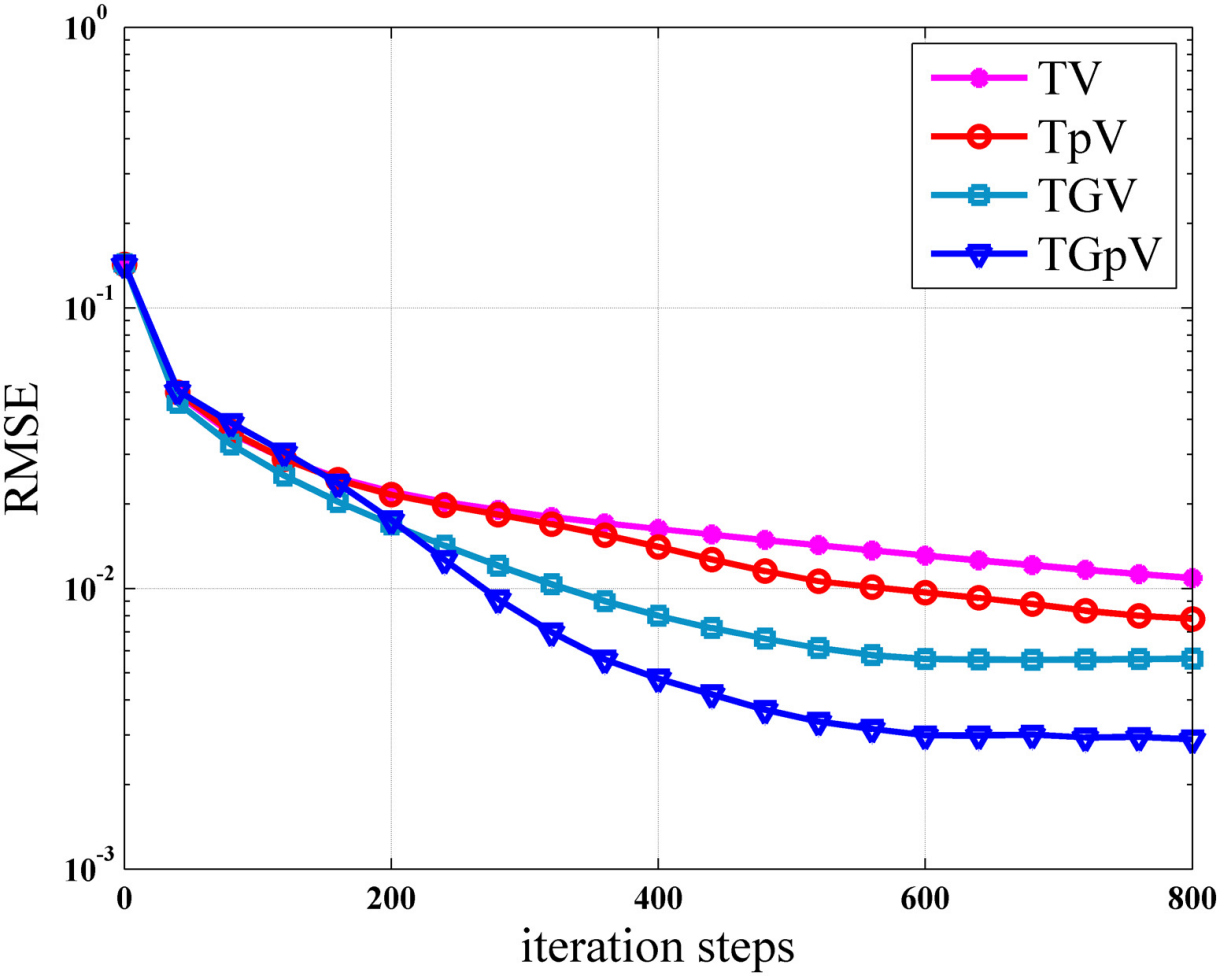
Root mean squared errors as a function of iterations for different methods in the noise-free case.

To further demonstrate the superiority of TGpV-ADM algorithm, the RMSE of the reconstructions from the different methods was calculated, and the calculation results are shown in Fig 12. The TGpV-ADM algorithm could converge to a steady status and is obviously more accurate and effective over the other methods. The RMSE, PSNR and NRMSD of each reconstruction method are listed in Table 12. Compared with other methods, TGpV-ADM method can visibly obtain more accurate images.

**Table 12:** Evaluations of the results reconstructed by different methods from noise-free projection dataset in digital CS-phantom studies.

	RMSE	PSNR	NRMSD
TV-ADM	1.0883e-02	39.2649	2.9532e-02
TpV-ADM	7.7744e-03	42.1866	2.1096e-02
TGV-ADM	5.6228e-03	45.0009	1.5258e-02
TGpV-ADM	2.8992e-03	50.7543	7.8672e-03

### Noisy cases

In the simulation of noisy cases, the noise level *N*_0_ was set to 1×10^6^. Meanwhile, the parameters should also be correspondingly adjusted.*e* was set to 10^−5^. The parameter settings of ADM framework in the four algorithms were listed as follows: *μ* and *λ*_0_ were set to 64 and 32, respectively, and *τ* was set to 1.3. For the TGV-ADM and TGpV-ADM methods, *α*_0_, *α*_1_, and *λ*_1_ were set to 1, 1, and 32, respectively. For the TpV and TGpV algorithms, *p* was set to 0.9. The number of iterations for each reconstruction was 150.

**Fig 13:**
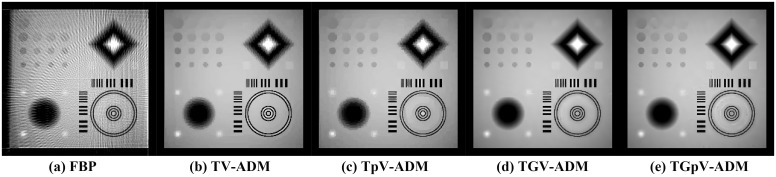
Image reconstruction of the CS-phantom from noisy projection dataset. Results of the (a) FBP, (b)TV-ADM, (c) TpV-ADM, (d) TGV-ADM, and (e) TGpV-ADM minimization methods. Display window is [0.1, 0.6].

**Fig 14:**
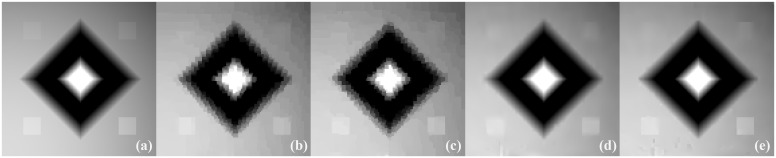
Reconstructed ROIs of Quadrant I from noisy projection dataset. From left to right are (a) original image, results of the (b) TV-ADM, (c) TpV-ADM, (d) TGV-ADM, and (e) TGpV-ADM minimization methods. Display window is [0.2, 0.55].

**Fig 15:**
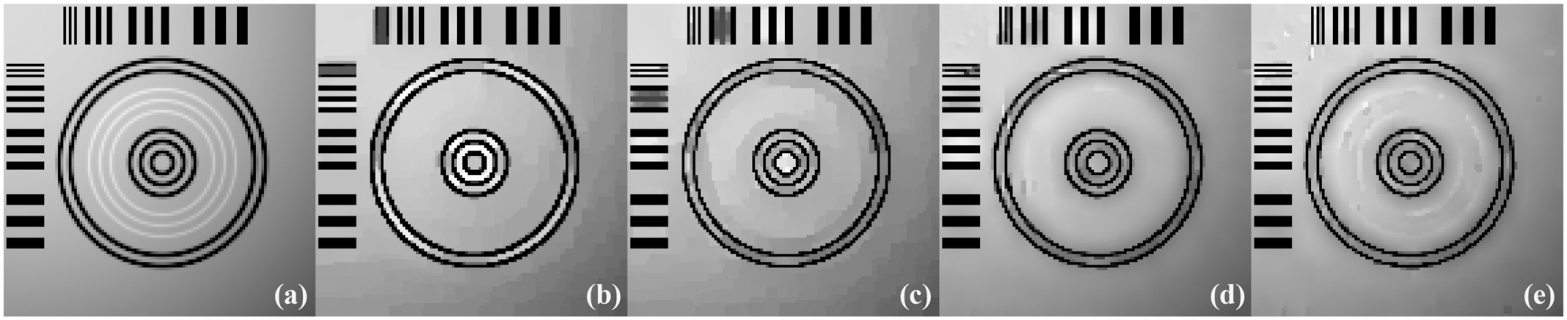
Reconstructed ROIs of Quadrant IV from noisy projection dataset. From left to right are (a) original image, results of the (b) TV-ADM, (c) TpV-ADM, (d) TGV-ADM, and (e) TGpV-ADM minimization methods. Display window is [0.2, 0.55].

**Fig 16:**
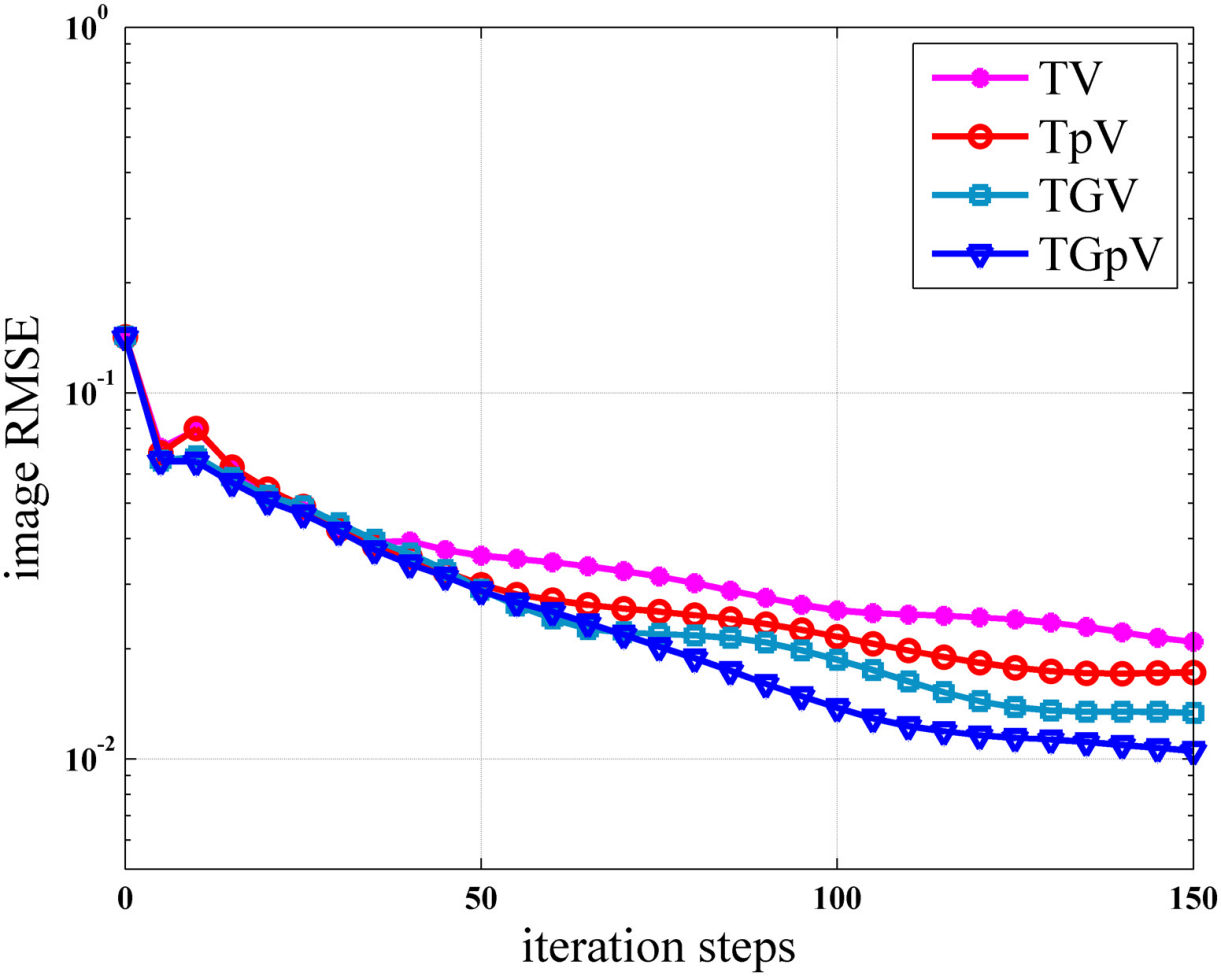
Root mean squared errors as a function of iterations for different methods in the noisy case.

Fig 13 shows the images reconstructed using the different methods, and the corresponding zoomed ROIs are shown in Figs 14 and 15. TGpV-ADM method still provides relatively better results than the other three methods in the noisy cases. To assess the performance of the proposed method quantitatively, the corresponding RMSE of the reconstructions is calculated and plotted in Fig 16. The RMSE, PSNR and NRMSD of each reconstruction method in noisy case are listed in Table 13. The proposed TGpV-ADM method successfully minimized the objective functions and effectively improved the image quality for noisy data.

**Table 13:** Evaluations of the results reconstructed by different methods from noisy projection dataset in digital CS-phantom studies.

	RMSE	PSNR	NRMSD
TV-ADM	2.0898e-02	33.6504	5.6366e-02
TpV-ADM	1.7254e-02	35.2623	4.6819e-02
TGV-ADM	1.3351e-02	37.4896	3.6229e-02
TGpV-ADM	1.0521e-02	39.5590	2.8549e-02

The average computation time of TV-ADM, TpV-ADM, TGV-ADM, and TGpV-ADM methods for digital CS-phantom studies are listed in Table 14. The table shows that the time consumption of TGpV-ADM method is slightly larger than that of the other methods. The present algorithm can preserve a good balance between performance and computation.

**Table 14:** Running time (in CPU seconds) of different methods in digital CS-phantom studies.

	TV-ADM	TpV-ADM	TGV-ADM	TGpV-ADM
Noise-free Cases	47.459	51.473	60.153	62.952
Noisy Cases	16.375	16.882	16.791	17.157

### Real data study

To demonstrate further the potential capability of the proposed method, we performed a radiological anthropomorphic head phantom (Chengdu Dosimetric Phantom, CPET Co. Ltd, Chengdu, China) [50] study from real data for clinical use. The phantom is shown in S3 Fig and the specification of it is described in ICRU Report 48 [51]. In this study, CT projection data were acquired using a CT scanner mainly comprising an X-ray source (Hawkeye130, Thales, France) and a flat-panel detector (Varian 4030E, USA). The tube voltage is set to 100 kVp. The x-ray tube current was set at 200 μA and the duration of x-ray pulse at each projection view was 180 ms during the acquisition. The central slice of the sinogram data was extracted for 2D investigation and was modeled with 820 bins on a 1D detector for 2D image reconstruction. The associated imaging parameters of the CT scanner were as follows: (1) 360 projection views were acquired evenly for a 360° rotation on a circular orbit, (2) the distance from the detector to the X-ray source was 1610 mm, (3) the distance from the rotation center to the source was 678 mm, and (4) the space of each detector bin was 0.508 mm. All of the reconstructed images comprised a 300 × 300 square of pixels. The size of each pixel was 0.585 mm × 0.585 mm. We evenly extracted a 120- and 180-view projection from the sinogram data for illustration purposes.

In the experiment, the algorithm parameters of all ADM frameworks were set to *μ* = 32, *λ*_0_ = 16, and *τ* = 1.3. *e* was set to 10^−5^. For the TGV-ADM and TGpV-ADM methods, *α*_0_, *α*_1_, and *λ*_1_ were set to 1, 1, and 16, respectively. For the TpV and TGpV algorithms, *p* was set to 0.9. The number of iterations for each reconstruction was 100.

**Fig 17:**
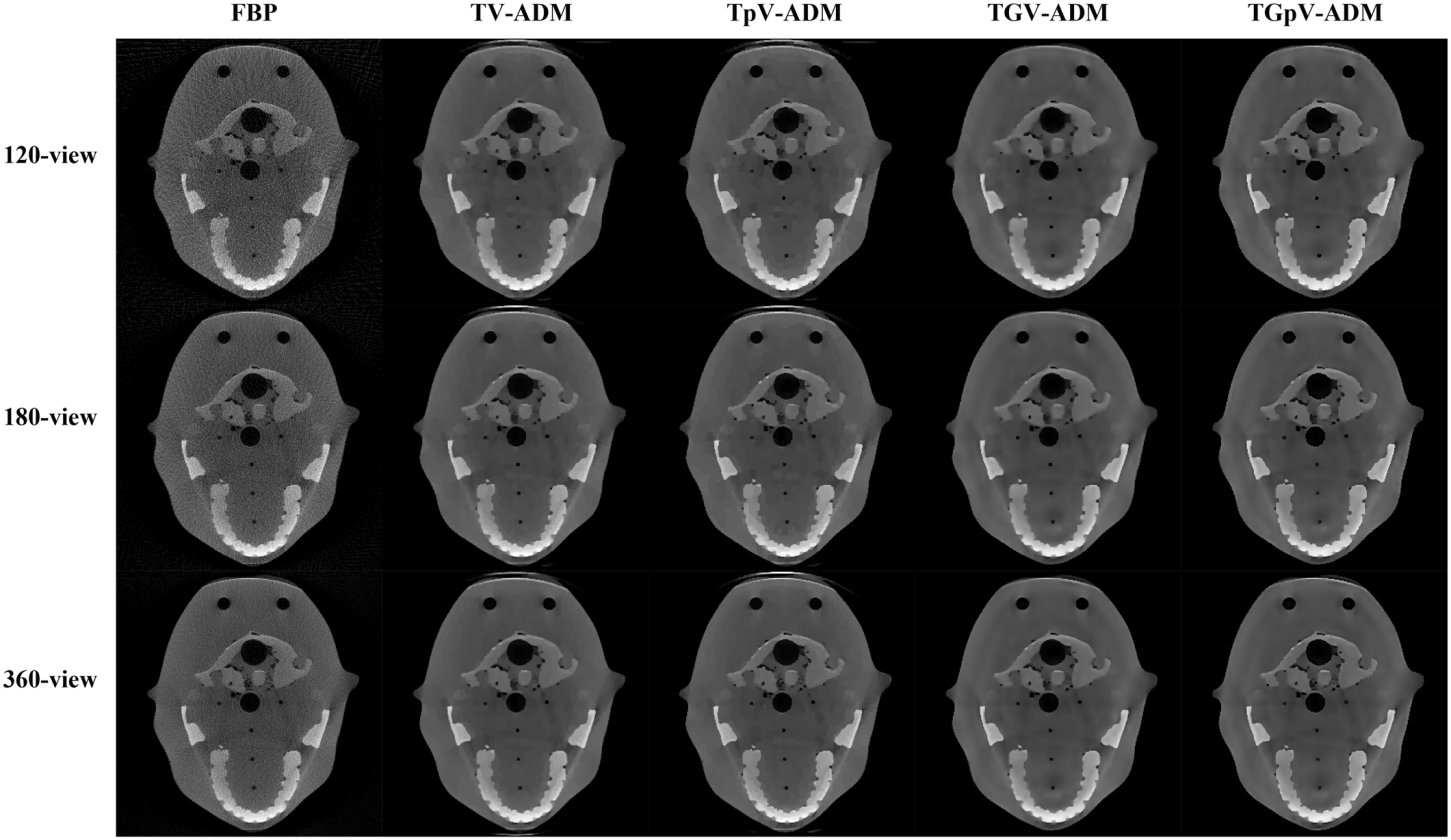
Images reconstructed using the FBP, TV-ADM, TpV-ADM, TGV-ADM, and TGpV-ADM methods from 120-, 180-, and 360-view projections, respectively. Rows from the top to the bottom are the reconstructed results from 120-, 180-, and 360-view projections, respectively. From left to right in each row, results of the FBP, TV-ADM, TpV-ADM, TGV-ADM, and TGpV-ADM methods are presented. Display window is [0.005, 0.0525] mm^−1^.

**Fig 18:**
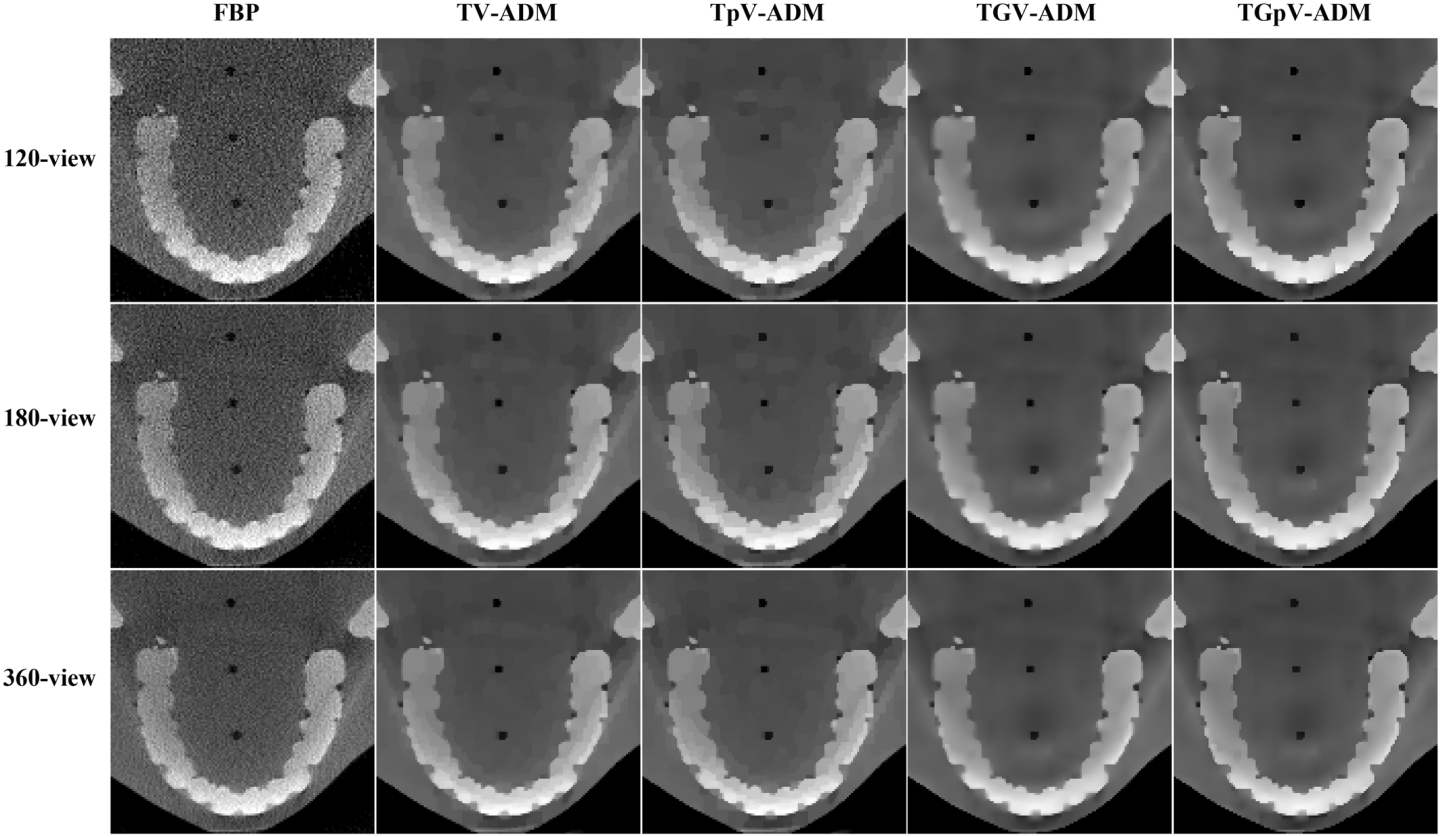
Zoomed-in views of images reconstructed using the FBP, TV-ADM, TpV-ADM, TGV-ADM, and TGpV-ADM methods from 120-, 180-, and 360-view projections, respectively. Rows from the top to the bottom are the reconstructed results from 120-, 180-, and 360-view projections, respectively. From left to right in each row, results of the FBP, TV-ADM, TpV-ADM, TGV-ADM, and TGpV-ADM methods are presented. Display window is [0.005, 0.0525] mm^−1^.

The reconstructed image results for the different methods from 120-, 180-, and 360-view projections are shown in Fig 17. The corresponding zoomed-in ROIs are shown in Fig 18. The TV-ADM and TpV-ADM methods have more patchy artifacts than the other two methods, and some details are oversmoothed in the reconstruction images. TGpV-ADM method exhibits remarkable advantages over the other methods in terms of detail preservation. Meanwhile, the results further suggest that compared with the other three methods, the TGpV-ADM method can achieve a more outstanding capability in dose reduction.

## Discussion

TV-based CT image reconstruction that employs the image gradient sparsity can reduce the X-ray sampling rate and remove the unwanted artifacts but may cause unfavorable oversmoothing and staircase effects under the piecewise constant assumption. TGV (a generalization of TV) involves high-order derivatives and is suited to regularize range images. The original TGV is based on an *l*_1_-norm expression. We introduced a TGpV model that considers the *l*_p_-norm and then developed an optimization-based reconstruction algorithm to extract additional sparsity information from the original TGV.

Compared with the other methods, the proposed TGpV-ADM method shows better image reconstruction results in both smoothly varying regions and sharp edges. Furthermore, the proposed method is robust to noise and shows much faster convergence than the other methods. There is small difference between the time consumption of the TGpV-ADM and TGV-ADM methods. On the one hand, with the increase of projection data, the proportion of projection/back-projection procedure will increase simultaneously. On the other hand, as the effective access to realizing high-performance computation of the subminimization problems by FFTs, the presented algorithm can keep a good and stable performance of balancing the accuracy and efficiency with the increase in computational scale.

Multiple parameter settings are likely to be involved in any reconstruction design and can significantly influence reconstruction results. Reconstruction under different parameter settings is likely to yield different levels of image quality. In the study, even when *p* = 0.9, which is very close to *p* = 1.0, the gains from the TGpV-ADM method are outstanding compared with those from the TGV-ADM method. To guide an adequate adaptation of the image reconstruction task, reconstructions using different parameters are given in S2 Appendix. Although we cannot provide the “best” selection strategy, the suggested metrics employing TGpV minimization allows high-quality image recovery with sparse projection data and suggests a clinically useful potential for radiation dose reduction.

The framework and metrics are only considered for the 2D fan-beam cases. We hope to extend the model to cone-beam CT and to investigate effective graphics processing unit-based implementation to gain significant improvement with minimal computational cost. Addressing this question is one of our future research focuses.

## Conclusion

In this paper, we present a TGpV regularization model to adaptively preserve the edge information while avoiding the staircase effect for few-view CT image reconstruction. The new model is solved by splitting variables with an efficient alternating minimization scheme. All of the subproblems have efficient solutions after using generalized p-shrinkage mappings and partial Fourier transform. Experimental results show that the proposed TGpV-ADM method can reconstruct sharp edges accurately and smoothly varying image regions from insufficient data. In particular, the proposed method shows considerable advantages over the standard TGV-ADM and TpV-ADM algorithms.

## Supporting Information

S1 Appendix Implementations of TV-ADM, TpV-ADM, and TGV-ADM algorithms. (DOC)

S2 Appendix Experimental results with different parameters. (DOC)

S1 Fig A typical Moby phantom used in the simulation study. Display window is [0, 1].
(TIF)

S2 Fig A typical CS-phantom used in the simulation study. Display window is [0, 1].
(TIF)

S3 Fig An anthropomorphic head phantom used in the real data study. (TIF)
