# Supplementary material for: Constrained Total Generalized p-Variation Minimization for Few-View X-Ray Computed Tomography Image Reconstruction
Source: PLoS One. 2016 Feb 22;11(2):e0149899. doi: 10.1371/journal.pone.0149899 (PMC4764011; doi:10.1371/journal.pone.0149899)
Supplement: S1 Appendix — (DOC) [file pone.0149899.s001.doc]

**S1 Appendix. Implementations of TV-ADM, TpV-ADM, and TGV-ADM algorithms.**

**1. TV-ADM algorithm**

To solve the linear system of Eq. (4), a constrained TV minimization model for describing the intensity variations of an image is used as follows:

$\text{ }u^{*}=\arg\mathop{\text{min}}\limits_{u} \left\|\nabla u\right\|_{1},\text{ }subject to\text{ }\left\|Au-b\right\|_{2}\leq e,$ (A1)

where $e$ is a data-error tolerance parameter.

By introducing a vector $z$, we consider the following constrained minimization problem, which is equivalent to (A1):

$\begin{matrix} \arg\mathop{\text{min}}\limits_{z} \left\|z\right\|_{1}\text{ } \\ \text{s.t.}\left\{\begin{matrix} Au-b=\sigma,\text{ }\left\|\sigma\right\|_{2}\leq e \\ \nabla u=z \end{matrix}\right.. \end{matrix}$ (A2)

Next, we reformulate the constrained minimization problem as an unconstrained optimization task through an augmented Lagrangian method. The augmented Lagrangian energy associated to (A2) is defined as:

$\begin{matrix} L_{\text{ }A}=\mathop{\text{min}}\limits_{u,z} \left\|z\right\|_{1}+r_{0}^{T}(z-\nabla u)+\frac{\lambda_{0}}{2}\left\|z-\nabla u\right\|_{2}^{2} \\ \text{ }-r_{1}^{T}(Au-b-\sigma)+\frac{\mu}{2}\left\|Au-b-\sigma\right\|_{2}^{2} \end{matrix}$ (A3)

where $r_{0},r_{1}$ are Lagrange multipliers, and $\lambda_{0},\text{ }\mu$ are positive constants used to balance the terms.

The solution to minimize $L_{\text{ }A}$ can be approximated efficiently by the alternating direction method; at each iteration, an approximate minimizer is found with respect to the variables $u$ and $z$ and the Lagrange multipliers are updated alternately. Herein, we simply state the general scheme of TV-ADM in Algorithm A1.

| **Algorithm A1**: TV-ADM algorithm |
| --- |
| Input $A,b,\mu,\lambda_{0},\tau,e$, initialize $z^{0},u^{0}$, and $k=0$.  **While** “not converged,” **Do**  (1) $z$-subproblem: $z^{k+1}\leftarrow\max\left\{\left\Vert\nabla u^{k}-r_{0}^{k}/\lambda_{0}\right\Vert-1/\lambda_{0},0\right\}\cdot\text{sign}\left(\nabla u^{k}-r_{0}^{k}/\lambda_{0}\right)\text{ },\text{ }$   (2) $u$-subproblem: $J=Au-b-\sigma^{k}+r_{1}/\mu,$       (3) Update noise term $\sigma$ by: $\sigma^{k+1}\leftarrow\min\left\{1,\text{ }e/\left\Vert Au^{k+1}-b\right\Vert_{2}\right\}\cdot\left(Au^{k+1}-b\right),$   (4) Update multipliers by: $\begin{matrix} r_{0}^{k+1}\leftarrow r_{0}^{k}+\lambda_{0}(z^{k+1}-\nabla u^{k+1}), \\ r_{1}^{k+1}\leftarrow r_{1}^{k}+\mu(b+\sigma^{k+1}-Au^{k+1}), \end{matrix}$   (5)$k\leftarrow k+1$.  **End Do**  Obtain reconstruction result:$u$. |

**2. TpV-ADM algorithm**

The constrained TpV minimization model for CT image reconstruction can be described as follows:

$\text{ }u^{*}=\arg\mathop{\text{min}}\limits_{u} \left\|\nabla u\right\|_{p},\text{ }subject to\text{ }\left\|Au-b\right\|_{2}\leq e,$ (A4)

The theoretical derivation of the TpV-ADM algorithm is similar to TV-ADM algorithm except the derivation of z-subproblem. z-subproblem is solved by generalized p-shrinkage mappings. The general TpV-ADM algorithm scheme can be expressed as follows (List Algorithm A2).

| **Algorithm A2**: TpV-ADM algorithm |
| --- |
| Input $A,b,\mu,\lambda_{0},\tau,e$, initialize $z^{0},u^{0}$, and $k=0$.  **While** “not converged,” **Do**  (1) $z$-subproblem:    (2) $u$-subproblem: $J=Au-b-\sigma^{k}+r_{1}/\mu,$       (3) Update noise term $\sigma$ by: $\sigma^{k+1}\leftarrow\min\left\{1,\text{ }e/\left\Vert Au^{k+1}-b\right\Vert_{2}\right\}\cdot\left(Au^{k+1}-b\right),$   (4) Update multipliers by: $\begin{matrix} r_{0}^{k+1}\leftarrow r_{0}^{k}+\lambda_{0}(z^{k+1}-\nabla u^{k+1}), \\ r_{1}^{k+1}\leftarrow r_{1}^{k}+\mu(b+\sigma^{k+1}-Au^{k+1}), \end{matrix}$   (5)$k\leftarrow k+1$.  **End Do**  Obtain reconstruction result:$u$. |

**3. TGV-ADM algorithm**

The constrained TGV minimization model for CT image reconstruction can be described as follows:

$\arg\mathop{\text{min}}\limits_{u,\omega} \alpha_{0}\left\|\nabla u-\omega\right\|_{1}+\alpha_{1}\left\|\varepsilon(\omega)\right\|_{1},\text{ }s.t.\text{ }\left\|Au-b\right\|_{2}\leq e.\text{ }$ (A5)

The general TGV-ADM algorithm scheme can be expressed as follows (List Algorithm A3). For purposes of comparison, the Fourier-based solutions are also used in the implementations of $u$-subproblem and $\omega$-subproblem.

| **Algorithm A3**: TGV-ADM framework |
| --- |
| Input$A,b,\mu,\lambda_{0},\lambda_{1},\alpha_{0},\alpha_{1},\tau,e$, initialize$d^{0},s^{0},u^{0},\omega^{0}$, and$k=0$. Given $p\in(0,\text{ }1)$:  **While** “not converged,” **Do**  (1) Update $d^{k+1}$ by $d^{k+1}\leftarrow\max\left\{\left\Vert\nabla u^{k}-\omega^{k}-\frac{\widetilde{d}^{k}}{\lambda_{0}}\right\Vert-\alpha_{0}/\lambda_{0},0\right\}\cdot\text{sign}\left(\nabla u^{k}-\omega^{k}-\frac{\widetilde{d}^{k}}{\lambda_{0}}\right)\text{ },$   (2) Update $s^{k+1}$ by $s^{k+1}\leftarrow\max\left\{\left\Vert\varepsilon(\omega^{k})-\frac{\widetilde{s}^{k}}{\lambda_{1}}\right\Vert-\alpha_{1}/\lambda_{1},0\right\}\cdot\text{sign}\left(\varepsilon(\omega^{k})-\frac{\widetilde{s}^{k}}{\lambda_{1}}\right)\text{ },$   (3) Update $u^{k+1}$ by    (4) Update $\sigma^{k+1}$ by $\sigma^{k+1}\leftarrow\min\left\{1,\text{ }e/\left\Vert Au^{k+1}-b\right\Vert_{2}\right\}\cdot\left(Au^{k+1}-b\right),$   (5) Update $\omega^{k+1}$ by $\begin{matrix} \omega_{x}^{k+1}=\left(\lambda_{0}I+\lambda_{1}\nabla_{x}^{T}\nabla_{x}+\frac{\lambda_{1}}{2}\nabla_{y}^{T}\nabla_{y}\right)^{+} \\ \text{ }\left(\lambda_{0}(\nabla_{x}u^{k+1}-d_{x}^{k+1}-\frac{\widetilde{d}_{x}^{k}}{\lambda_{0}})+\lambda_{1}\nabla_{x}^{T}(s_{x}^{k+1}+\frac{\widetilde{s}_{x}^{k}}{\lambda_{1}})+\lambda_{1}\nabla_{y}^{T}(s_{z}^{k+1}+\frac{\widetilde{s}_{z}^{k}}{\lambda_{1}}-\frac{\nabla_{x}\omega_{y}^{k}}{2})\right), \end{matrix}$ $\begin{matrix} \omega_{y}^{k+1}=\left(\lambda_{0}I+\lambda_{1}\nabla_{y}^{T}\nabla_{y}+\frac{\lambda_{1}}{2}\nabla_{x}^{T}\nabla_{x}\right)^{+} \\ \text{ }\left(\lambda_{0}(\nabla_{y}u^{k+1}-d_{y}^{k+1}-\frac{\widetilde{d}_{y}^{k}}{\lambda_{0}})+\lambda_{1}\nabla_{y}^{T}(s_{y}^{k+1}+\frac{\widetilde{s}_{y}^{k}}{\lambda_{1}})+\lambda_{1}\nabla_{x}^{T}(s_{z}^{k+1}+\frac{\widetilde{s}_{z}^{k}}{\lambda_{1}}-\frac{\nabla_{y}\omega_{x}^{k+1}}{2})\right). \end{matrix}$     (6) Update multipliers by $\begin{matrix} \widetilde{d}^{k+1}\leftarrow\widetilde{d}^{k}+\lambda_{0}(d^{k+1}-\nabla u^{k+1}+\omega^{k+1}), \\ \widetilde{s}^{k+1}\leftarrow\widetilde{s}^{k}+\lambda_{1}(s^{k+1}-\varepsilon(\omega^{k+1})), \\ \widetilde{r}^{k+1}\leftarrow\widetilde{r}^{k}+\mu(b+\sigma^{k+1}-Au^{k+1}), \end{matrix}$   (7)$k\leftarrow k+1$.  **End Do**  Obtain reconstruction result:$u$. |
