# Supplementary material for: Constrained Total Generalized p-Variation Minimization for Few-View X-Ray Computed Tomography Image Reconstruction
Source: PLoS One. 2016 Feb 22;11(2):e0149899. doi: 10.1371/journal.pone.0149899 (PMC4764011; doi:10.1371/journal.pone.0149899)
Supplement: S2 Appendix — (DOC) [file pone.0149899.s002.doc]

**S2 Appendix. Experimental results with different parameters.**

In this section we discuss the impacts of different parameters of TGpV algorithm on the reconstructed image quality for sparse-view CT image reconstruction. Though parameters , and are used to balance the two regularization terms, they often are set to be equal in many applications [26]. The balance between the first and second derivatives is often adjusted by the positive weights $\alpha_{0}$ and $\alpha_{1}$ of TGpV regularization term. Thus, this section focus the investigation on comparing the results reconstructed with different weights $\alpha_{0}$ and $\alpha_{1}$, and different order $p$.

**1. Digital Moby phantom Study**

In the first group of experiments, a digital Moby phantom is used. The imaging configuration and reconstruction settings are same with the noisy case 2. First, we compare the reconstruction results with different weights $\alpha_{0}$ and $\alpha_{1}$, the images reconstructed with different weights are shown in Fig. A1. The profiles of the images along the 52th horizontal rows are shown in Fig. A2.

The results suggest that the reconstructions show better quality with the selection of $\alpha_{0}=1,\alpha_{1}=2$ and $\alpha_{0}=1,\alpha_{1}=4$. In the results reconstructed with parameters $\alpha_{0}=2,\alpha_{1}=1$, some blocky artifacts are appeared. $\alpha_{0}>\alpha_{1}$ means that the specific weight of first derivative is larger than that of the second derivative. Thus, a larger $\alpha_{0}$ may lead to staircase effects in the images. However, a larger $\alpha_{1}$ may also lead to a decrease on the image quality. It may result some wavy artifacts in the smooth region. This phenomenon could be observed in the results reconstructed with parameters $\alpha_{0}=1,\alpha_{1}=8$.

| 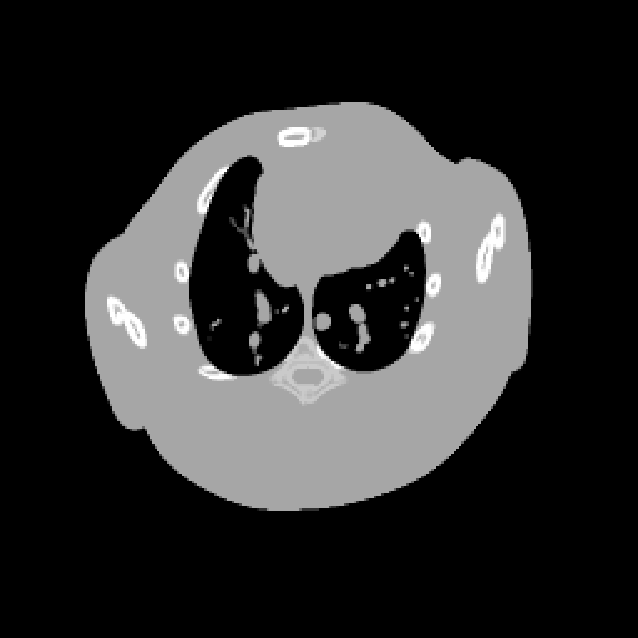 | 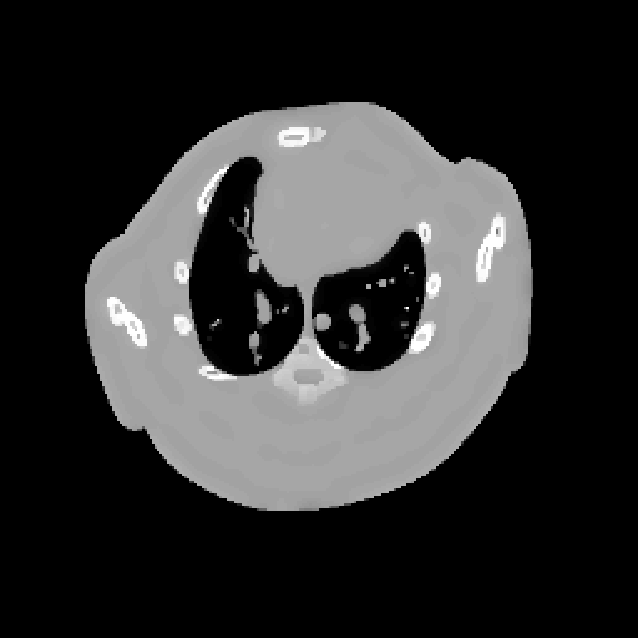 | 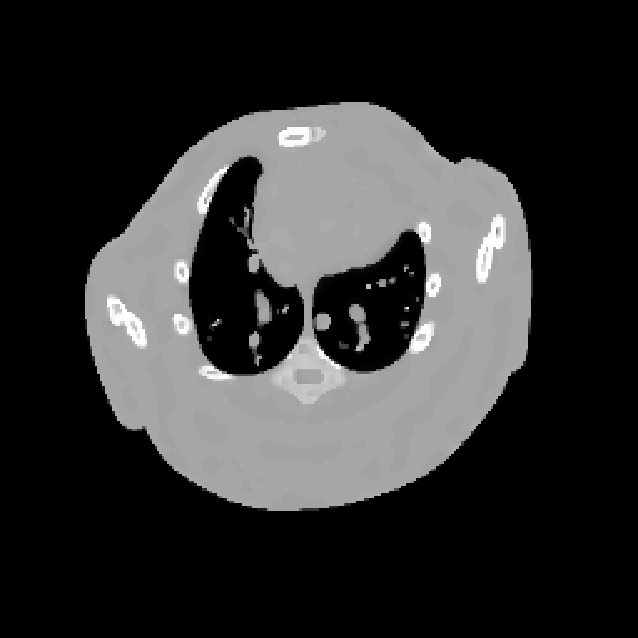 |
| --- | --- | --- |
| (a) original image | (b) $\alpha_{0}=2,\alpha_{1}=1$ | (c) $\alpha_{0}=1,\alpha_{1}=1$ |
| 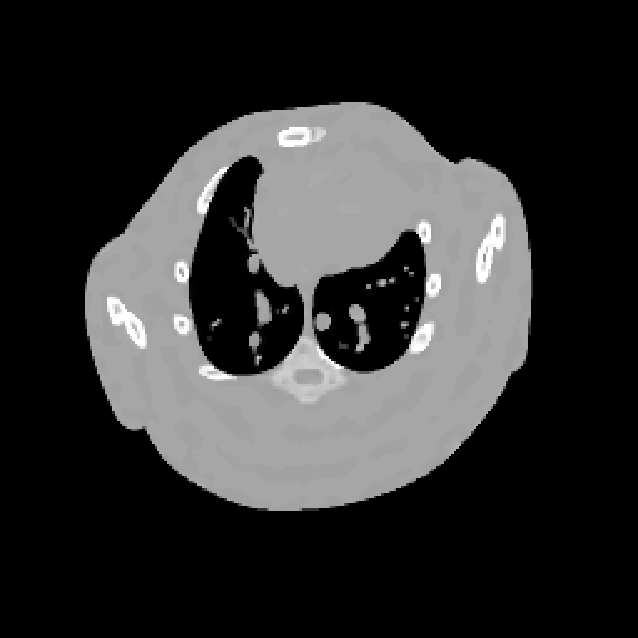 | 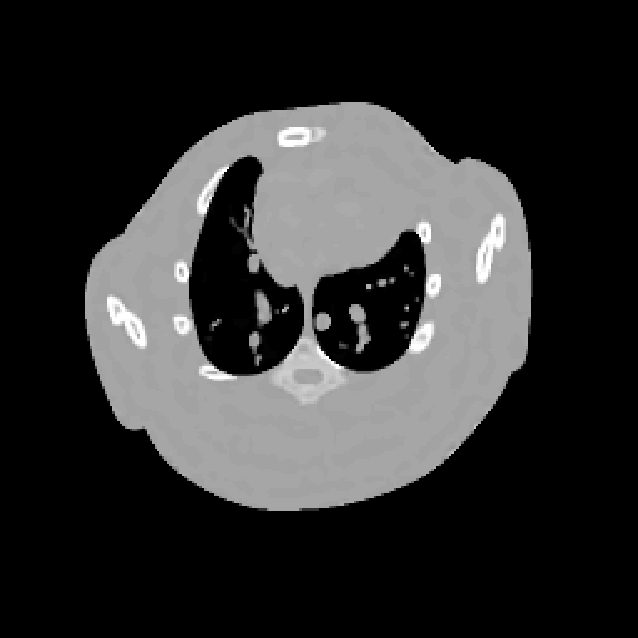 | 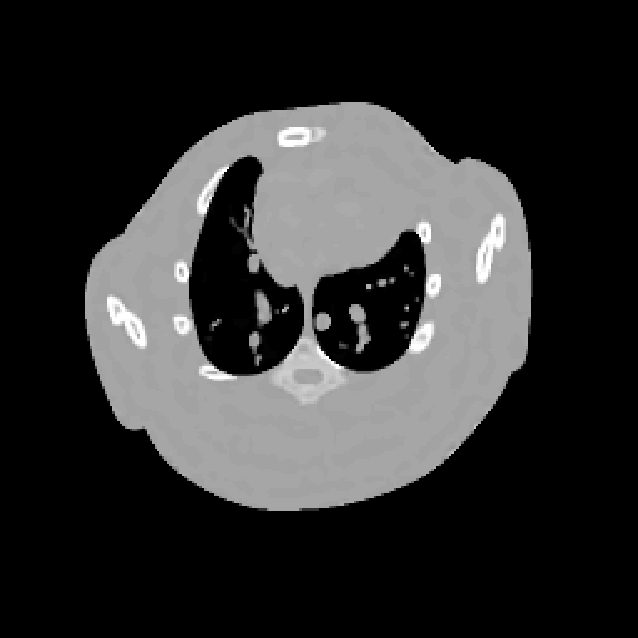 |
| (d) $\alpha_{0}=1,\alpha_{1}=2$ | (e) $\alpha_{0}=1,\alpha_{1}=4$ | (f) $\alpha_{0}=1,\alpha_{1}=8$ |

**Fig. A1. Image reconstruction of the Moby phantom from noisy projection dataset with different weights (*p*=0.9).** Display window is [0.2, 1.0].

|  | |
| --- | --- |
| (a) $\alpha_{0}=2,\alpha_{1}=1$ | |
|  |  |
| (b) $\alpha_{0}=1,\alpha_{1}=1$ | (c) $\alpha_{0}=1,\alpha_{1}=2$ |
|  |  |
| (d) $\alpha_{0}=1,\alpha_{1}=4$ | (e) $\alpha_{0}=1,\alpha_{1}=8$ |

**Fig. A2. Horizontal profiles (52th row) in the reconstruction results of the Moby phantom from noisy projection dataset with different weights.**

The parameter $p$ also plays a key role in the algorithm. The images reconstructed with different values of $p$ are shown in Fig. A3. The profiles of the images along the 52th horizontal rows are shown in Fig. A4. Generally, a smaller value of $p$ often performs better for images with geometric shapes. However, there are various inconsistencies in data acquisition, a too small value of $p$ may result the loss of some subtle details. In the results, the selection of a small $p$ could obtain more sharp edges in the image, but the accuracy of image may not be the best.

| 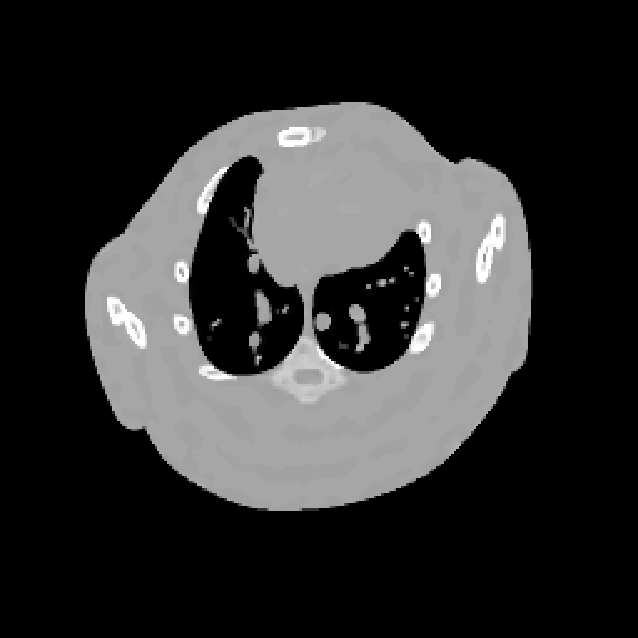 | 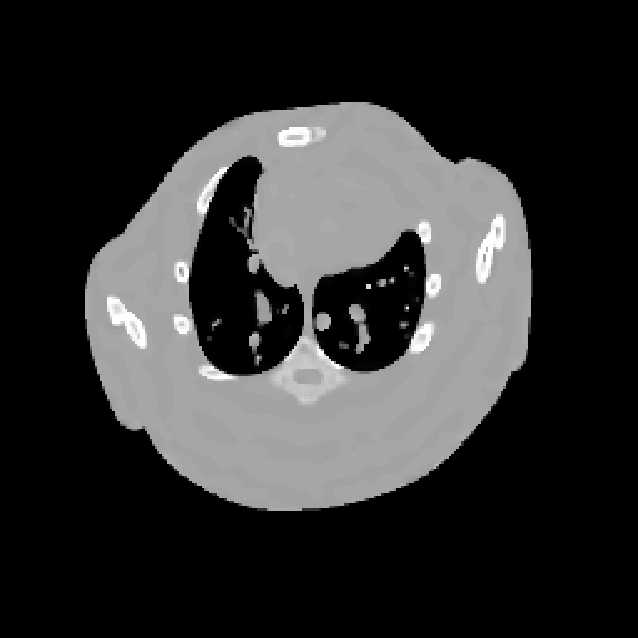 |
| --- | --- |
| (a) *p*=0.9 | (b) *p*=0.75 |
| 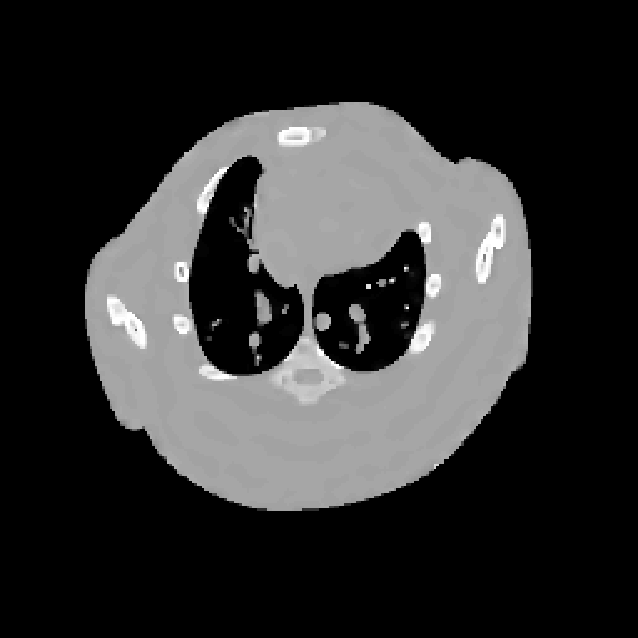 | 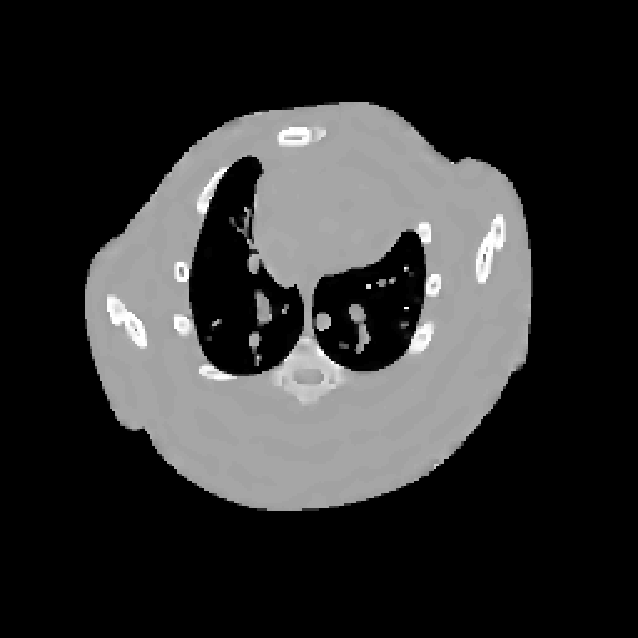 |
| (c) *p*=0.6 | (c) *p*=0.45 |

**Fig. A3. Image reconstruction of the Moby phantom from noisy projection dataset with different values of *p* (**$\alpha_{0}=1,\alpha_{1}=2$**).** Display window is [0.2, 1.0].

|  |  |
| --- | --- |
| (a) *p*=0.9 | (b) *p*=0.75 |
|  |  |
| (c) *p*=0.6 | (c) *p*=0.45 |

**Fig. A4. Horizontal profiles (52th row) in the reconstruction results of the Moby phantom from noisy projection dataset with different values of p.**

**2. Real data study**

In the second group of study, a radiological anthropomorphic head phantom is used. The imaging configuration and reconstruction settings are same with the real data study. The number of projection view is 120.

The images reconstructed with different weights $\alpha_{0}$, $\alpha_{1}$ and the corresponding zoomed-in ROIs are shown in Fig. A5. Again, a larger $\alpha_{0}$ will lead to a solution with staircase effects, and a larger $\alpha_{1}$ appears visually to achieve a wavy and blurred result. The images reconstructed with different values of $p$ are shown in Fig. A6. It can be seen that a smaller value of $p$ could achieve a better result with sharp geometric shapes.

|  |  |  |
| --- | --- | --- |
|  |  |  |
| (a) $\alpha_{0}=2,\alpha_{1}=1$ | (b) $\alpha_{0}=1,\alpha_{1}=1$ | (c) $\alpha_{0}=1,\alpha_{1}=2$ |

**Fig. A5. Images reconstructed of the anthropomorphic head phantom from 120-view projections with different weights (*p*=0.8).** Display window is [0.005, 0.0525] mm^−1^.

| 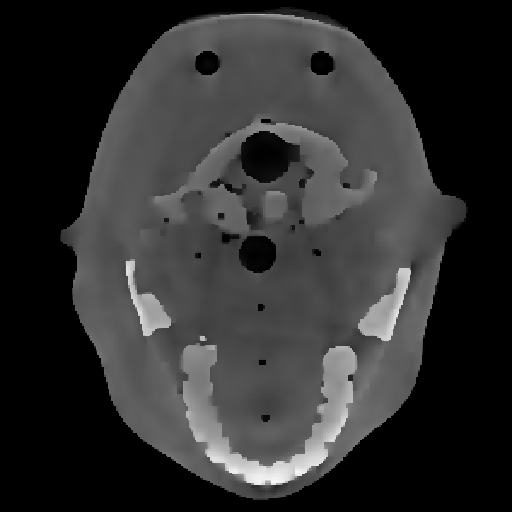 |  |  |
| --- | --- | --- |
| 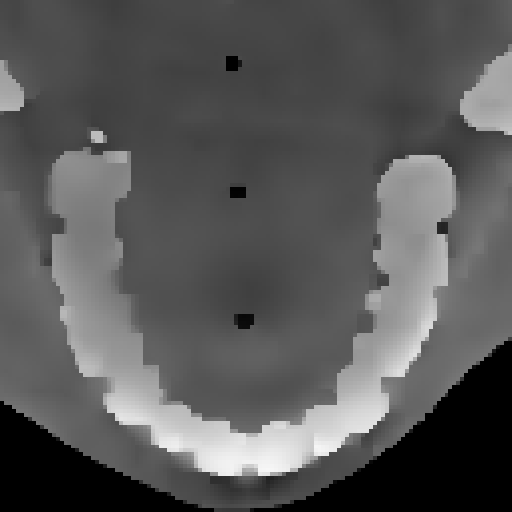 |  |  |
| (a) *p*=0.9 | (b) *p*=0.7 | (c) p=0.5 |

**Fig. A6. Images reconstructed of the anthropomorphic head phantom from 120-view projections with different values of *p* (**$\alpha_{0}=1,\alpha_{1}=1$**)** Display window is [0.005, 0.0525] mm^−1^.
