# Supplementary figures and images for: Constrained Total Generalized p-Variation Minimization for Few-View X-Ray Computed Tomography Image Reconstruction
Source: PLoS One. 2016 Feb 22;11(2):e0149899. doi: 10.1371/journal.pone.0149899 (PMC4764011; doi:10.1371/journal.pone.0149899)

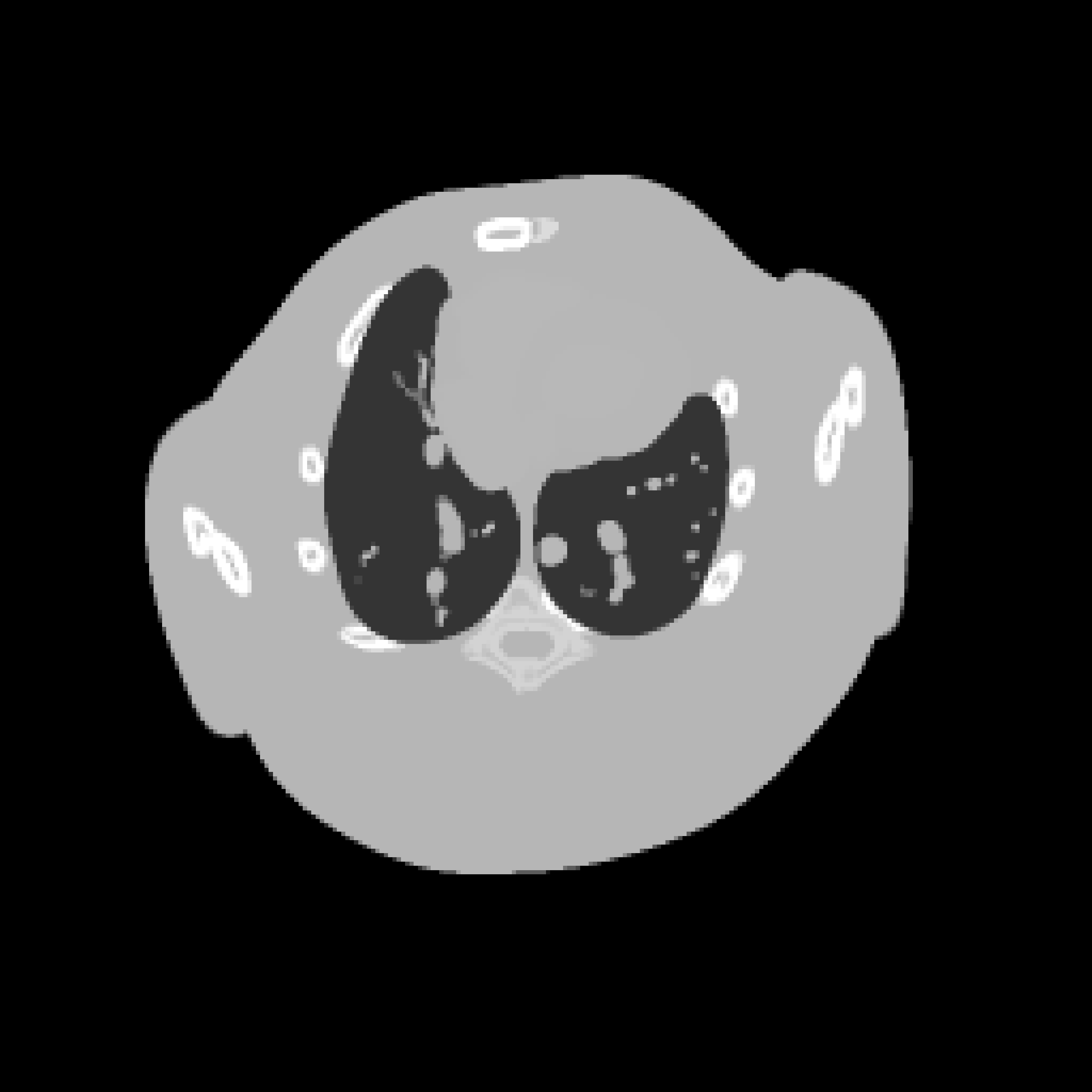

Supplement: S1 Fig — Display window is [0, 1]. (TIF) [file pone.0149899.s003.tif]

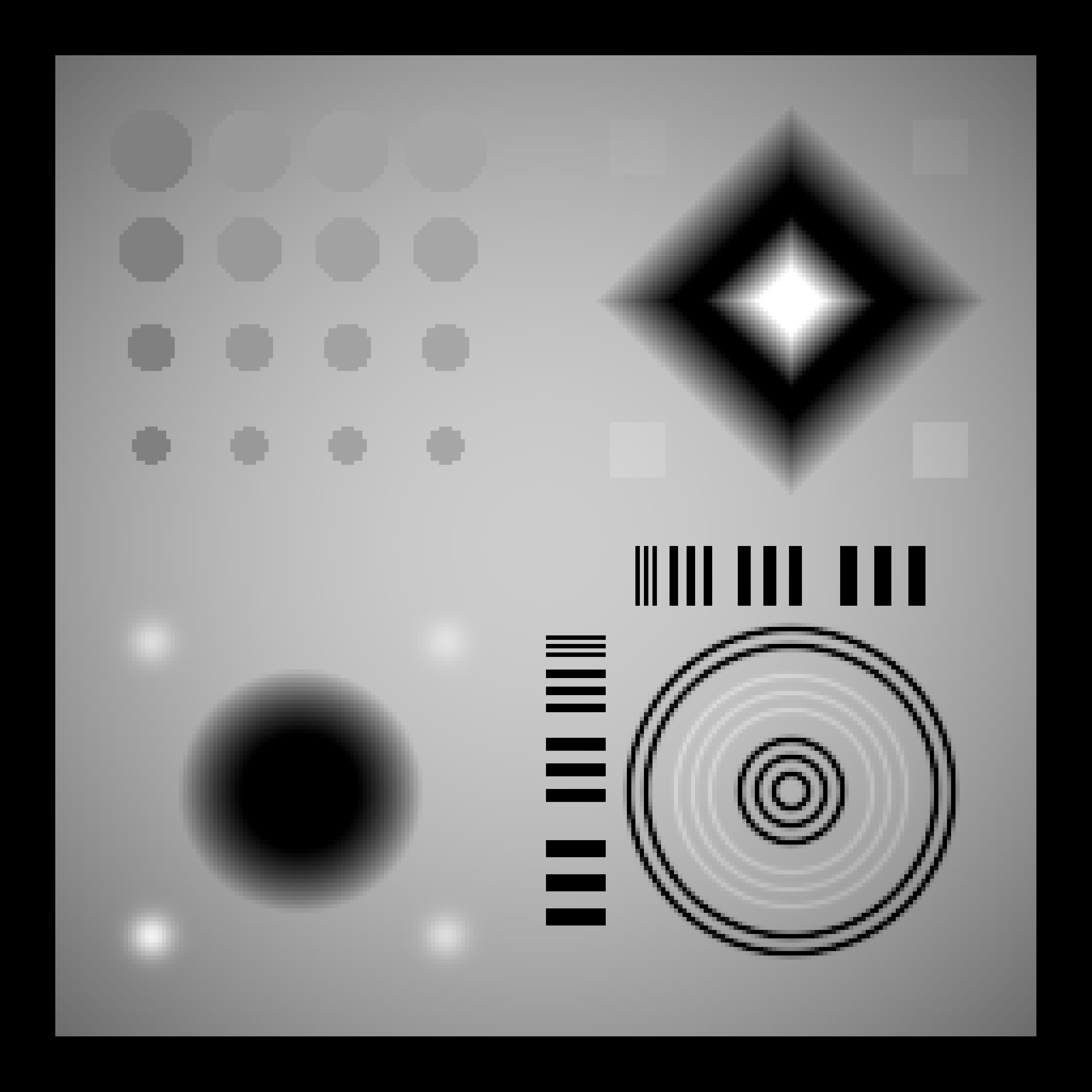

Supplement: S2 Fig — Display window is [0, 1]. (TIF) [file pone.0149899.s004.tif]

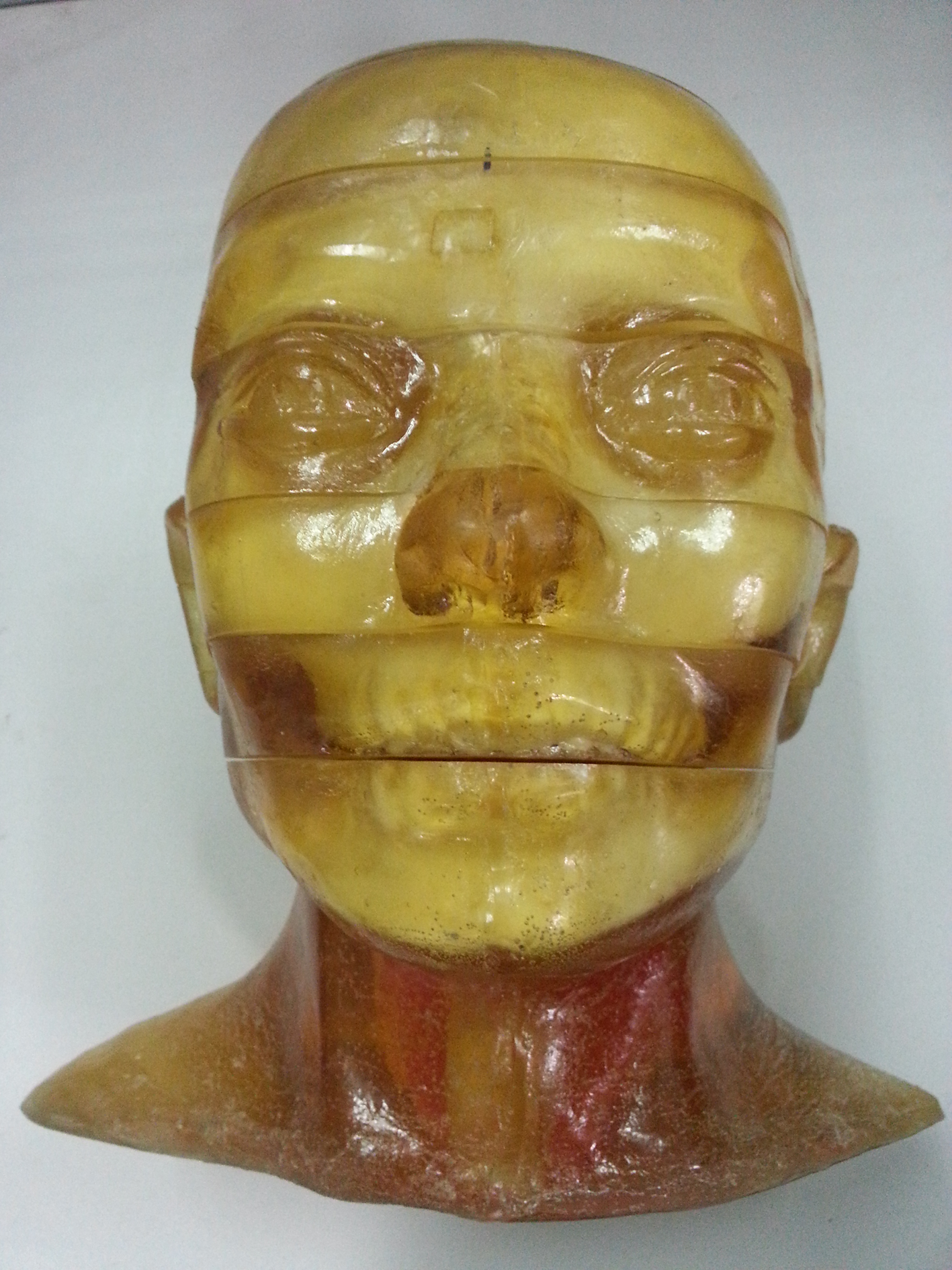

Supplement: S3 Fig — (TIF) [file pone.0149899.s005.tif]
